# SlideCNA: spatial copy number alteration detection from Slide-seq-like spatial transcriptomics data

**DOI:** 10.1186/s13059-025-03573-y

**Published:** 2025-05-02

**Authors:** Diane Zhang, Åsa Segerstolpe, Michal Slyper, Julia Waldman, Evan Murray, Robert Strasser, Jan Watter, Ofir Cohen, Orr Ashenberg, Daniel Abravanel, Judit Jané-Valbuena, Simon Mages, Ana Lako, Karla Helvie, Orit Rozenblatt-Rosen, Scott Rodig, Fei Chen, Nikhil Wagle, Aviv Regev, Johanna Klughammer

**Affiliations:** 1 Broad Institute of MIT and Harvard, Cambridge, MA USA; 2 Massachusetts Institute of Technology, Cambridge, MA USA; 3 Department of Medical Oncology, Dana-Farber Cancer Institute, Boston, MA USA; 4 Department of Microbiology, Immunology, and Genetics, Faculty of Health Sciences, Ben-Gurion University, Beersheba, Israel; 5 Gene Center and Department of Biochemistry, Ludwig-Maximilians-Universität München, Munich, Germany; 6 Department of Pathology, Brigham and Women’s Hospital, Boston, MA USA; 7 Klarman Cell Observatory, Broad Institute of MIT and Harvard, Cambridge, MA USA; 8 Genentech, 1 DNA Way, South San Francisco, CA USA

**Keywords:** Spatial transcriptomics, Slide-seq, Single-cell RNA-seq, Copy number alterations, Cancer, Tumor microenvironment, Clonality

## Abstract

Solid tumors are spatially heterogeneous in their genetic, molecular, and cellular composition, but recent spatial profiling studies have mostly charted genetic and RNA variation in tumors separately. To leverage the potential of RNA to identify copy number alterations (CNAs), we develop SlideCNA, a computational tool to extract CNA signals from sparse spatial transcriptomics data with near single cellular resolution. SlideCNA uses expression-aware spatial binning to overcome sparsity limitations while maintaining spatial signal to recover CNA patterns. We test SlideCNA on simulated and real Slide-seq data of (metastatic) breast cancer and demonstrate its potential for spatial subclone detection.

**Supplementary Information:**

The online version contains supplementary material available at 10.1186/s13059-025-03573-y.

## Background

The spatial organization of tumors at the genetic, expression, and histological levels provides important insights into a tumor’s evolution, growth, microenvironment, and response to therapy [1]. In spot-based spatial transcriptomics methods such as Visium-ST [2] and Slide-seq [3], a tissue section is positioned on a glass slide coated with RNA capture probes or beads, such that polyadenylated RNA molecules are spatially barcoded in an untargeted, arrayed fashion. Recent advances have led to increasingly higher resolution (55 µm in Visium-ST and 10 µm in Slide-seq), albeit at the cost of higher sparsity [4, 5]. Spatial profiling studies have provided insights into the cellular and molecular organization of tumor tissue ecosystems, but—aside from direct experimental measurements [6]—these have not yet been associated with the genetic state of malignant cells, including tumor cell clonality. Relating the spatial context of clonal genetic events to cell states in the tumor ecosystem can help distinguish malignant and non-malignant cells and shed light on the impact of the microenvironment on the mutational landscape, therapeutic resistance, progression, and metastasis.

InferCNV is a computational method designed to infer genomic copy number alterations (CNAs) from single-cell RNA-seq (scRNA-seq) profiles [7], and it has since been applied to Visium-ST data of tumor tissues for spatial CNA detection [8]. Similarly, CopyKAT is a more recent computational tool to detect CNAs from scRNA-seq data that uses expression smoothing and a Gaussian mixture model to infer a diploid reference population, and Bayesian segmentation to find CNA breakpoints [9]. However, methods such as InferCNV and CopyKAT were developed for data at single-cell resolution, not spot-based spatial profiling, where transcripts from adjacent cells of different clones or types may be captured on one barcode. Additionally, the relatively sparse read counts of higher-resolution, high-density spot-based spatial profiling methods like Slide-seq (as opposed to Visium-ST) poses a challenge for these existing CNA detection methods, which were developed for less sparse scRNA-seq data. Lastly, the spatial information has the potential to provide insight into subclonal structure and contextualize CNA events, which current methods do not leverage.

## Results and discussion

**Fig. 1:**
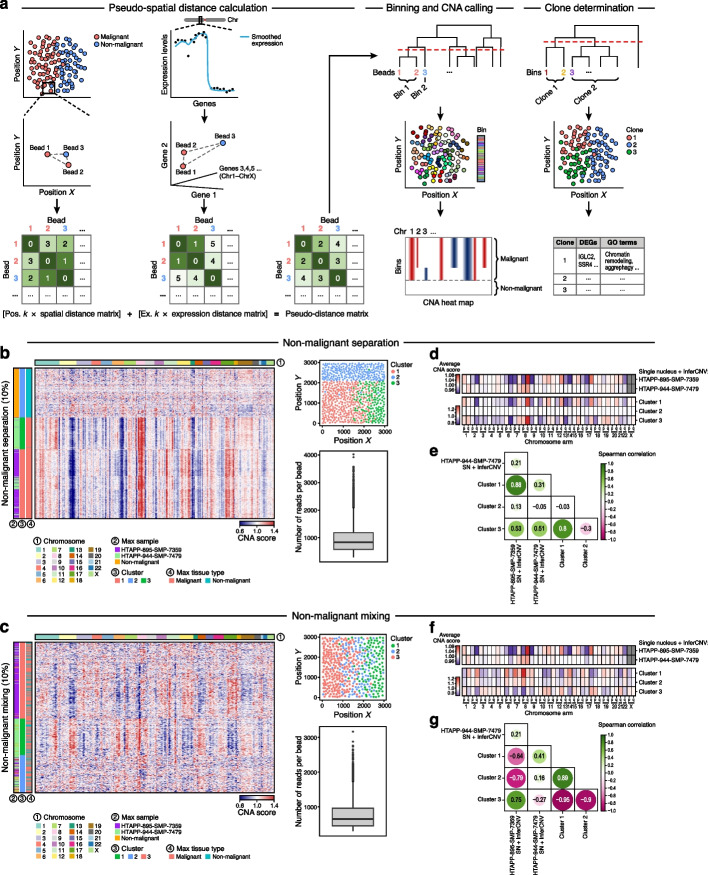
SlideCNA methodology schematic and benchmarking on in silico Slide-seq-like data generated from snRNA-seq data of two MBC biopsies. **a** SlideCNA methodology involves calculating bead distances by a combined spatial- and expression-based pseudo-distance, binning beads based on pseudo-distance, and determining malignant clones based on CNA profiles. SlideCNA heat map (amplification > 1, deletion < 1), spatial plot of bins colored by assigned cluster, and boxplot of number of reads per bin after filtering for beads with > 300 counts across all genes for the in silico non-malignant-separated dataset (**b**) and non-malignant-mixed dataset (**c**) with UMI counts downsampled to 10%. Comparison of average SlideCNA CNA scores per chromosome arm of each SlideCNA-defined cluster for in silico data with non-malignant separation (**d**) and mixing (**f**) with UMI counts downsampled to 10% to the InferCNV profiles from the original snRNA-seq data. Pairwise Spearman correlation of CNA profiles as in **d** and **f** for in silico data with non-malignant separation (**e**) and mixing (**g**) with UMI counts downsampled to 10%

To address this challenge, we develop SlideCNA to improve CNA inference and clone calling in higher-resolution, high-density spot-based spatial transcriptomics data by leveraging the increased spatial resolution and density of Slide-seq data with a spatio-molecular binning step to address signal sparsity (Fig. 1a, Methods). Similar to InferCNV, SlideCNA implements an expression-based smoothing approach across each chromosome with a weighted pyramidal average scheme. Then, SlideCNA adjusts all expression values by the average expression of a user-defined set of reference beads (or spots) for each gene and centers these values to produce relative smoothed expression intensities. To overcome Slide-seq’s expression sparsity, SlideCNA uses a spatial binning step to combine neighboring beads into bins that increase the signal (counts), while maintaining spatial structure. To this end, SlideCNA computes bead-by-bead distances in both expression and physical space, then takes a weighted linear combination of the expression and spatial distance matrices and hierarchically clusters this combined pseudo-distance matrix to group beads with similar expression profiles that are also proximal in physical space. SlideCNA partitions the beads into bins with a user-defined maximum number of beads per bin, calculates bin expression intensities as an average across the constituent beads, and normalizes and scales these intensities for UMI count to generate CNA scores.

We first tested SlideCNA’s ability to recover a ground truth CNA profile using two in silico (simulated) Slide-seq datasets with known clonal structure, which we constructed from (1) single-nucleus RNA-seq (snRNA-seq) data from two independent metastatic breast cancer (MBC) biopsies generated as part of the Human Tumor Atlas Pilot Project (HTAPP [10]) and (2) published scRNA-seq data from two independent primary breast cancer biopsies [11] (Additional file 1: Fig. S1a). Mimicking the process of RNA capture on Slide-seq beads for a tissue section, we placed 20,000 cell/nucleus profiles on a 2-dimensional square, positioning malignant cells of each sample on opposite regions in a gradient-like manner, such that cells of both samples would be equally mixed in the middle (Additional file 1: Fig. S1b, bottom label). Non-malignant cell/nucleus profiles were either positioned (1) separately but adjacent to the malignant cell/nucleus profiles, reflecting a discrete compartmentalized normal population (Additional file 1: Fig. S1b, left and S1c), or (2) randomly across the square, reflecting a more challenging intermixing of non-malignant and malignant cells (*e.g.,* due to infiltration) (Additional file 1: Fig. S1b, right, and S1d). For each scenario, we applied a Gaussian kernel to create a square of 10,000 beads with combined expression values of the constituent cell/nucleus profiles (or their fractions) (Additional file 1: Fig. S1b, bottom). To roughly approximate the sparsity of Slide-seq data and test sensitivity, we downsampled UMI counts of the in silico beads to 25%, 10%, and 2%, with 10% corresponding to the typical UMI counts per bead in real Slide-seq data.

Applied to the HTAPP MBC in silico datasets, SlideCNA generated CNA profiles with unique patterns in two malignant clusters, recapitulating the original positioning of single nucleus profiles, both in the normal separation and normal mixing case (Fig. 1b–e, Additional file 1: Fig. S1e,f). In the normal separation and mixing cases, we observed consistent results without downsampling, and at 25% and 10% downsampling, but not with 2% downsampling (Additional file 1: Figs. S2 and S3). Similarly, applied to the published breast cancer in silico datasets [11], SlideCNA resolved the two artificial malignant clones, down to 10% downsampling, in both the normal separation and normal mixing cases, albeit to a lesser extent in the latter (Additional file 1: Figs. S4, S5, and S6). Notably, the CNA profiles derived for each in silico “clone” were consistent with those generated for the constituent sc/snRNA-seq data by InferCNV (Spearman’s *ρ* = 0.41–0.88) (Fig. 1d–g). Thus, SlideCNA can potentially recover subclones with distinct CNA profiles in Slide-seq-like data.

**Fig. 2:**
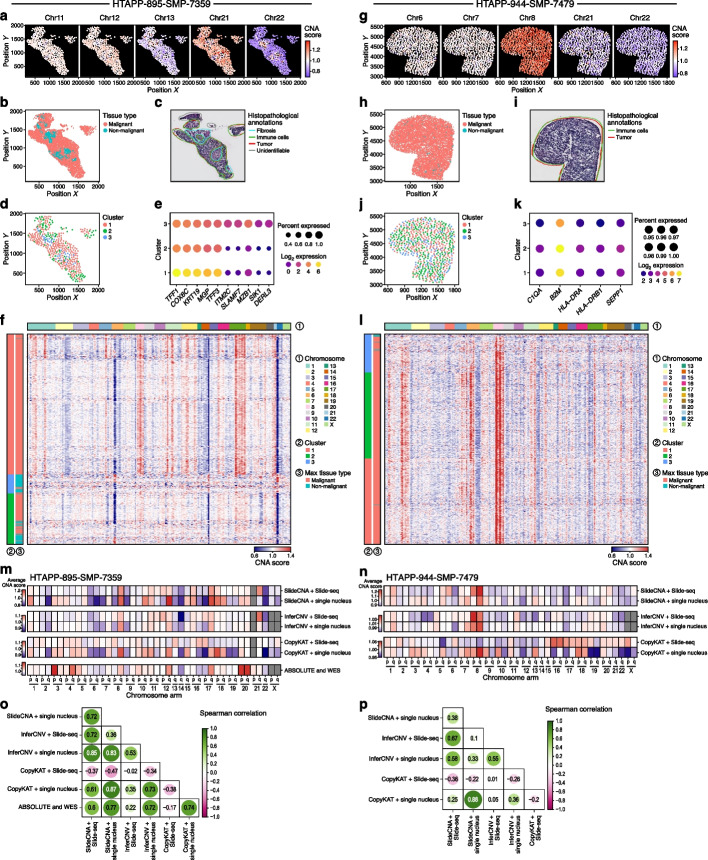
SlideCNA identifies spatial CNA patterns in Slide-seq MBC samples. **a**–**f** refer to sample HTAPP-895-SMP-7359 and **g**–**l** refer to sample HTAPP-944-SMP-7479. **a**, **g** Spatial plot of beads colored by mean CNA score across the indicated chromosomes, selected to demonstrate a range of spatial CNA patterns. **b**, **h** Spatial plot of beads annotated as non-malignant (blue) or malignant (pink) with non-malignant beads serving as reference for SlideCNA. **c**, **i** H&E stains of consecutive sections matching the Slide-seq samples with histopathological annotations. **d**, **j** Spatial plot of binned beads colored by SlideCNA-defined cluster designation. **e**, **k** Top DEGs for each cluster detected from the SlideCNA profile. DEGs were colored by average log_2_ cluster expression and sized by the percent of beads expressing that gene in the cluster (negative binomial generalized linear model *p*-adj < 0.05). **f**, **l** SlideCNA heat map of malignant and non-malignant binned beads annotated with cluster assignment. Comparison of average SlideCNA, InferCNV, CopyKAT, and ABSOLUTE CNA scores per chromosome arm for Slide-seq, snRNA-seq, and WES data for HTAPP-895-SMP-7359 (**m**) and HTAPP-944-SMP-7479 (**n**). Pairwise Spearman correlation of CNA profiles as in **m** and **n** for HTAPP-895-SMP-7359 (**o**) and HTAPP-944-SMP-7479 (**p**)

Next, we applied SlideCNA to two real MBC Slide-seq samples matching the snRNA-seq data used for the in silico analysis after annotating (by cell type annotation transfer from the snRNA-seq data) non-malignant beads as a reference against which contiguous chromosomal expression changes could be scored to infer CNAs (Methods). In the first tumor (HTAPP-895-SMP-7359), chromosome-wide CNA scores were spatially heterogeneous (Fig. 2a–c). Chromosomes 13, 21, and 22 showed strong CNA signals (deletion for chromosomes 13 and 22 and amplification for chromosome 21), specifically in regions annotated as malignant, while chromosomes 11 and 12 consistently did not show CNAs across space. Malignant beads partitioned into two clusters that mapped to different regions by their inferred CNA profile, with some significant differentially expressed genes (DEGs) between clusters (TFF1&3, COX6 C, KRT19, MGP) (Fig. 2d–f, clusters 1 and 2). Non-malignant beads occupied their own spatially distinct cluster as well (Fig. 2d, cluster 3). Notably, the spatial position of malignant and non-malignant beads corresponded overall to expert pathologist annotation of a hematoxylin and eosin (H&E) stain of an immediately adjacent section (Fig. 2a–c). In the second tumor (HTAPP-944-SMP-7479), we recovered two malignant bead clusters with few DEGs, similar CNA patterns, and weak spatial variability in CNAs (e.g., chromosomes 8, 21, and 22), but distinct from the non-malignant reference population (Fig. 2g–l). The weak differences between the two malignant clusters may not qualify as sufficient evidence for biologically meaningful subclones (consistent with the snRNA-seq data), but rather showcase the possibility to define clones based on weak signals, which should then be assessed for plausibility based on the accompanying SlideCNA visualizations.

To evaluate SlideCNA’s consistency across replicates, we applied it to two additional MBC Slide-seq datasets (HTAPP-878-SMP-7149 and HTAPP-880-SMP-7179) with matching snRNA-seq data (Additional file 1: Figs. S7 and S8). Each dataset had two replicates from serial sections showing substantial agreement, both spatially and in a cell profile embedding graph, which did not show signs of batch effects (Additional file 1: Fig. S7a,f). To quantify similarity between replicate SlideCNA inferred CNA profiles, we calculated the pairwise Spearman’s rank correlation based on average CNA scores per chromosomal arm. Replicates were processed independently for comparison showing overall consistency in CNA profiles (*ρ* = 0.9–0.94) with some variability due to differences in sample quality, i.e., number of covered beads (Additional file 1: Fig. S7), as well as jointly (through spatial alignment) for further analysis and benchmarking, showing consistent CNA profiles with those obtained for the individual replicates (*ρ* = 0.91–0.99) (Additional file 1: Figs. S7 d,e,i,j and S8). These results highlight SlideCNA’s consistency across replicates, and, through the joint analysis, robustness to identifying batch (section)-specific clusters.

Reassuringly, the SlideCNA-inferred profiles for all four Slide-seq samples were largely consistent with those obtained from the matching snRNA-seq data called with either InferCNV, CopyKAT, or SlideCNA with non-spatial parameters (Additional file 1: Figs. S1e,f, S9c,d, S10,c,d, and S11a). To quantify this visual consistency, for each sample, we calculated the pairwise Spearman’s rank correlation between each pair of method (SlideCNA, InferCNV, CopyKAT)-modality (Slide-seq, snRNA-seq) combination based on average CNA scores per chromosomal arm (Fig. 2m–p, Additional file 1: Fig. S8o–r). These correlations largely confirmed the consistency of SlideCNA’s inferred Slide-seq CNA profiles with InferCNV (*ρ* = 0.58–0.88) and CopyKAT (*ρ* = 0.25–0.65) inferred snRNA-seq CNA profiles except for sample HTAPP-880-SMP-7179, for which CopyKAT results were discordant to those of InferCNV (*ρ* = − 0.42) and SlideCNA (*ρ* = − 0.39). CNA profiles for Slide-seq generated by InferCNV (*ρ* = 0.42–0.67) or CopyKAT (*ρ* = −0.38–0.22) were generally less correlated to the snRNA-seq derived profiles. SlideCNA also uniquely identified several arm-level CNAs not found when applying InferCNV or CopyKAT to Slide-seq, such as CNAs on chromosomes 8p, 9p, and 16q for HTAPP-895-SMP-7359, chromosomes 1q and 6q for HTAPP-944-SMP-7479, and chromosomes 1p, 5q, 16q, 19q, and 20 for HTAPP-880-SMP-7179, all of which were present in the snRNA-seq data (Fig. 2m–n and Additional file 1: Figs. S8m–n, S9a,b, S10a,b, and S11b). This highlights SlideCNA’s unique ability to consistently detect subchromosomal CNAs in Slide-seq data while preserving the spatial landscape of CNAs and overcoming signal sparsity.

As further genomic validation, for HTAPP-895-SMP-7359, we also had access to matching whole exome sequencing (WES) data (from a different biopsy of the same metastatic site) and obtained CNA profiles with ABSOLUTE [12]. Here, CNA profiles from ABSOLUTE analysis on WES were largely consistent with those of snRNA-seq data with SlideCNA (*ρ* = 0.77), InferCNV (*ρ* = 0.72), and CopyKAT (*ρ* = 0.74) and Slide-seq data with SlideCNA (*ρ* = 0.6) but not with InferCNV (*ρ* = 0.22) nor CopyKAT (*ρ* = −0.17) (Fig. 2m–p). Consistent CNAs included the chr2q, 6q, 14q, and 22q deletions and chr8q, 12q, and 18p amplifications, while partial discordance may be explained by allelic differences resolved by WES but not RNA-seq (Additional file 1: Fig. S9e), further supporting SlideCNA’s accuracy.

Finally, we tested SlideCNA’s performance on cell-type resolved spatial transcriptomics data by applying a “bead decomposition,” where each bead’s expression profile is computationally decomposed into its constituent cell-type specific expression profiles [13]. Applying this approach to the HTAPP-895-SMP-7359 and HTAPP-944-SMP-7479 Slide-seq datasets resulted in 23,103 and 38,900 decomposed beads and a median of 47 and 50 reads per decomposed bead, respectively. In each sample, SlideCNA identified strong CNA profiles in the malignant population consistent with those detected in the original Slide-seq data but with substantially improved CNA signal and cluster assignment—especially for the sample with highly intermixed malignant and non-malignant cell types (HTAPP-944-SMP-7479) (Additional file 1: Fig. S12a,b). However, bead decomposition comes at the cost of potentially introducing biases such as over-representation and over-contrasting of cell types that usually constitute minorities within a bead, which we observe in the large and non-homogeneous non-malignant populations of both samples (Additional file 1: Fig. S12a iii and b iii). Therefore, we suggest bead decomposition as a promising enhancement of SlideCNA, but not as the standard approach.

## Conclusions

We demonstrated that SlideCNA simultaneously captures spatial signal and identifies CNAs from Slide-seq data, with potential power to detect subclonal structure, despite limited capture efficiency and imperfect separation between normal and malignant expression. Compared to existing methods, SlideCNA is well-suited for spatially dense, transcriptionally sparse methods like Slide-seq where the spatial binning approach is most effective. While SlideCNA performs best with spatially segregated reference cell populations, it can also handle highly intermixed non-malignant beads, albeit with some loss in CNA signal. In such cases, bead decomposition shows promise to further improve the signal. Finally, limitations include that SlideCNA requires whole transcriptome data, operates better on spatially dense methods such as Slide-seq and the upcoming Visium HD as opposed to the popular Visium-ST platform, and is not suitable for methods with limited gene-panels such as current Merfish or Xenium or methods with non-count expression values such as Codex. Overall, SlideCNA should help empower studies of tumor ecosystems in large-scale spatial CNA screening and expression analysis in clinical samples.

## Methods

### Tissue samples

Matching snRNA-seq and Slide-seq data were generated for four breast cancer liver metastases (HTAPP-895-SMP-7359 and HTAPP-944-SMP-7479 for the main analysis and HTAPP-878-SMP-7149 and HTAPP-880-SMP-7179 with two replicates each for supplemental analysis) of four different patients (Additional file 1: Fig. S1a). Tissues were collected as described previously [14]. Specifically, MBC OCT-frozen samples were obtained from the Center for Cancer Precision Medicine Bank. Fresh samples (core-needle biopsies) were frozen in optimal cutting temperature compound (OCT, Tissue-Tek Sakura). Cores were pre-coated with OCT by putting a thin layer of OCT down in the cryomold before placing an individual core in the center of the OCT mold in a straight line and adding additional OCT to fill the cryomold. The cryomold was then placed on dry ice for 5–15 min until the block was opaque before storing it at − 80 °C.

### Single nucleus RNA-seq

SnRNA-seq was performed as described previously [14]. Specifically, frozen tissue was placed on ice and in one well of a plate (Stem Cell Technologies, cat. no. 38015) and 1 ml of TST buffer was added to the well. Tissue was kept on ice and cut into pieces with Noyes Spring Scissors (Fine Science Tools, cat. no. 15514–12) for 10 min. Tissue mixture was filtered through a 40-µm Falcon cell strainer (ThermoFisher Scientific, cat. no. 08–771 - 1). The well was washed and filtered with 1 ml of detergent buffer solution, and 3 ml of 1 × ST buffer was added to a total well volume of 5 ml. The solution was centrifuged in a 15-ml Eppendorf tube for 5 min at 500* g* and 4 °C in a swinging bucket centrifuge. Pellet was resuspended in 1 × ST buffer, with a resuspension volume of 100–200 µl based on pellet size. The single nucleus suspension was filtered through a 35-µm Falcon cell strainer (Corning, cat. no. 352235). Ten thousand nuclei were selected with a C-chip disposable hemocytometer (VWR, cat. no. 82030–468) and transferred to Chromium chips for the Chromium Single Cell 3′ Library (V3, PN- 1000075) per manufacturer’s instructions (10 × Genomics).

### Slide-seq

Slide-seq was performed as previously described [5]. Specifically, core biopsies embedded in OCT (Tissue-Tek Sakura) and kept at − 80 °C were sectioned at 10 μm thickness onto one Slide-seq puck each (Additional file 1: Fig. S13a). Loaded Slide-seq pucks were incubated with hybridization buffer (6 × SSC with 2 U/μl RNase inhibitor (Lucigen, 30,281)) and subjected to first-strand cDNA synthesis (1 × Maxima RT buffer, 1 mM of each dNTP, 0.05 U/μl RNase inhibitor (Lucigen, 30,281), 2.5 µM Template switch oligo (TSO) and 10 U/μl Maxima H Minus reverse transcriptase (ThermoFisher, EP0742)) followed by tissue digestion (200 mM Tris–Cl pH 7.5, 400 mM NaCl, 4% SDS, 10 mM EDTA with 1:50 proteinase K (New England BioLabs, P8107S)), as previously described. Puck beads were released into suspension and washed in wash buffer (10 mM Tris pH 8, 1 mM EDTA, 0.01% Tween- 20) before incubation with exonuclease I (1 × ExoI buffer with 10 U/μl exonuclease I (New England BioLabs, M0293L)) to prepare for second-strand cDNA synthesis which was performed using the following reaction mix: 1 × Maxima RT buffer, 1 mM of each dNTP, 10 μM dN-SMRT oligonucleotide, and 0.125 U/μl Klenow enzyme (NEB, M0210). The sample was subjected to PCR amplification with 1 × Terra Direct PCR mix buffer, 2 μl Terra polymerase (Takara, 639,270), 2 μM TruSeq PCR handle primer, and 2 μM SMART PCR primer (98 °C for 2 min; four cycles of 98 °C for 20 s, 65 °C for 45 s and 72 °C for 3 min; 11 cycles of 98 °C for 20 s, 67 °C for 20 s and 72 °C for 3 min; 72 °C for 5 min; hold at 4 °C). cDNA was purified using AMPure XP beads and quantified on a Bioanalyzer High Sensitivity DNA chip (Agilent, 5067–4626) and on a Qubit high sensitivity dsDNA kit (Invitrogen, Q32851). Six hundred picograms of cDNA was tagmented with a Nextera XT kit (Illumina, FC- 131–1096) and libraries were indexed with TruSeq5 and the N700 series barcoded index primers. Libraries were purified using AMPure XP beads and sequenced on an Illumina NextSeq High Output flow cell with the settings: read1 44 bases, read2 39 bases, and index1 8 bases.

### snRNA-seq data pre-processing

Raw counts matrices were generated from fastq files using the 10x CellRanger pipeline as implemented in Cumulus [15], set to include reads from intronic regions and using the hg38 reference genome together with the corresponding gene annotation. SnRNA-seq raw count data were filtered and genes with ≤ 300 counts across all nuclei were removed. The filtered count matrix was transformed into a Seurat v4 [16] object through CreateSeuratObject, which involves calculating the percentage of mitochondrial transcript contamination with PercentageFeatureSet (default parameters), natural log normalization with a scale factor of 10,000 with NormalizeData, identifying variable genes with FindVariableFeatures (default parameters), scaling normalized counts while regressing out the number of RNA counts and percentage of mitochondrial genes with ScaleData, running PCA with 50 principal components with RunPCA, calculating *k*-nearest neighbors (KNN) with *k* = 20 neighbors to construct a shared nearest neighbors (SNN) graph with FindNeighbors, and identifying clusters based on the SNN with FindClusters (default parameters) [17, 18].

### Slide-seq data pre-processing

Count matrices and bead positions were generated using the Slide-seq pipeline (https://github.com/MacoskoLab/slideseq-tools) and the hg19 reference genome together with the corresponding gene annotation. To remove spurious measurements that did not originate from the tissue, Slide-seq data and matching H&E images were semi-manually aligned based on the tissue silhouettes and all measurements that did not overlap the tissue were removed. Low-quality beads were conservatively removed such that the fraction of beads with less than 100 counts did not exceed 35%. The Slide-seq count matrix and metadata were processed into a Seurat v4 object with the same steps described for snRNA-seq (above), which was then used to designate beads as normal or malignant (below) [3].

### De novo reference (non-malignant) cell selection in snRNA-seq

SingleR v.1.8.0 [19] was used to annotate snRNA-seq profiles using the Human Primary Cell Atlas (HPCA) [20], ENCODE [21], and BLUEPRINT [22] reference datasets by applying the functions HumanPrimaryCellAtlasData and BlueprintEncodeData from the R package celldex v1.11.1 [19]. Cell type annotations were unified to ensure comprehensive coverage using a cell unification list (NK: NK_cell, NK cells; Endothelial: Endothelial cells, Endothelial_cells; B: B-cells, B cell, B_cell; Macrophage: Macrophage, Macrophages; Chondrocyte: Chondrocytes; Monocyte: Monocyte, Monocytes; Neutrophil: Neutrophils; Fibroblast: Fibroblasts; Smooth muscle: Smooth muscle, Smooth_muscle_cells; Epithelial: Epithelial_cells, Epithelial cells). The endothelial, macrophage, natural killer (NK), and monocyte cells in each sample were selected as the non-malignant reference cells because other cell types are suspected to show strong localized expression (immune receptors of B and T cells), physiologically carry CNAs (hepatocytes), or could be misannotated malignant cells (fibroblasts), all of which could hamper the CNA quantification process (Additional file 1: Fig. S13 d). Non-malignant cells that were not used as reference were still included in the respective analysis to serve as internal controls.

### De novo reference bead selection in Slide-seq

Slide-seq beads were assigned cell types with Robust Cell Type Decomposition (RCTD) [23] using the matching snRNA-seq data together with their cell type annotations as reference and default parameters. The doublet_mode parameter was set to “full” to obtain a full representation of cell types captured in a bead. Finally, for each bead, the majority cell type was selected as the final annotation. To have a sufficient number of reference cells, all cell types other than MBC and MBC_neuronal were selected as the non-malignant reference cells.

### SlideCNA

To transform raw counts into gene expression intensities that reflect copy number changes, a modified version of the InferCNV processing pipeline [7] was employed. Initial removal of non-tissue-aligning beads and filtration of low-quality beads (see “Slide-seq data pre-processing” section) was followed by log_2_(TPM + 1) transformation of the raw counts matrix. Each gene was centered by subtracting its mean log_2_ expression in the reference beads. Relative expression intensities were capped at ≤ 3 and ≥ −3 and mitochondrial and Y chromosome genes were removed.

Genes were ordered by chromosome (using a gene-position file obtained from https://data.broadinstitute.org/Trinity/CTAT/cnv/hg38_gencode_v27.txt), and the centered expression intensities for each bead were smoothed across genomic regions in each chromosome by a pyramidal weighted moving average (default window of *k* = 101 or *k* = *n* if *n* < 101, with *n* = number of genes on the chromosome), with higher weights corresponding to closer genes. Thus, the smoothed expression (${\rm X}_{i}$) of each gene at position *i* (${g}_{i}$) was calculated as a weighted sum (${W}_{i}$) of its *m* nearest genes on both sides (${G}_{i}$). For genes at the ends of chromosomes, if there were less than *m* genes on one side, the number of remaining genes was used instead of *m* for that side.

$$\begin{array}{c}m=\frac{k-1}{2}\end{array}$$

$$\begin{array}{c}S_i = \begin{cases}(m + 1 - i, \dots, m + 1, m, \dots, 2, 1), & \text{if } i \leq m \\(1, 2, \dots, m, m + 1, \dots, n - 1), & \text{if } i> n - m \\(1, 2, \dots, m, m + 1, m, \dots, 2, 1), & \text{otherwise}\end{cases}\end{array}$$

$$\begin{array}{c}{W}_{i}={S}_{i}\cdot \frac{1}{{\sum }_{x\in {S}_{i}}x}\end{array}$$

$$\begin{array}{c}G_i = \begin{cases}(g_1, \dots, g_i, g_{i+1}, \dots, g_{i+m-1}, g_{i+m}), & \text{if } i \leq m \\(g_{i-m}, g_{i-m+1}, \dots, g_{i-1}, g_i, \dots, g_n), & \text{if } i> n - m \\(g_{i-m}, g_{i-m+1}, \dots, g_{i-1}, g_i, g_{i+1}, \dots, g_{i+m-1}, g_{i+m}), & \text{otherwise}\end{cases}\end{array}$$

$$\begin{array}{c}{X}_{i}={W}_{i}\cdot {G}_{i}\end{array}$$

Each gene was re-centered by subtracting the mean-smoothed expression intensity of both malignant and non-malignant beads from each bead to create a baseline of zero expression intensity change. Then, the mean-smoothed expression intensity of reference beads was subtracted and the log_2_ transformation was reversed to rescale to expression intensities, resulting in a gene expression intensity matrix (beads × genes).

Following this expression smoothing, to address sparsity in Slide-seq data, multiple adjacent beads were binned together, separately for non-malignant and malignant beads, under the assumption that spatially proximal and expression-similar beads are likely to have similar CNAs. To quantify bead spatial proximities, a bead spatial distance matrix (${X}_{spatial}$, beads × beads) was calculated from the bead spatial matrix (beads × dimensions, where dimensions = 2 corresponding to the x and y axes) using Euclidean distance. To quantify how close beads are in expression space, the gene expression intensity distance matrix (${X}_{expression}$, beads × beads) was calculated from the gene expression intensity matrix (beads × genes) using Euclidean distance. Integrating these, beads were binned by pseudo-distance (${X}_{pseudo}$, beads × beads), defined as a linear combination of the bead spatial distance matrix (${X}_{spatial}$, beads × beads) and the gene expression intensity distance matrix (${X}_{expression}$, beads × beads). Both the distance matrix and relative gene expression intensity matrix were weighted by the adjustable parameters ${k}_{spatial}$ and ${k}_{expression}$, respectively. ${k}_{spatial}$ = 55 and ${k}_{expression}$ = 1 were used here to prioritize binning beads that are spatially close together:

$${X}_{pseudo}={k}_{spatial}\cdot {X}_{spatial}+{k}_{expression}\cdot {X}_{expression}$$

The stability of ${k}_{spatial}$ and ${k}_{expression}$ values were assessed by bootstrapping (Additional file 1: Fig. S14).

When running SlideCNA on a non-spatial dataset (e.g., snRNA-seq), the parameter spatial was set to False and ${k}_{spatial}$ and ${X}_{spatial}$ were omitted from the pseudospatial distance calculation. Bins were plotted across space for visual inspection. The resulting pseudo-distance matrix of all beads was hierarchically clustered using Ward’s hierarchical agglomerative clustering method [24] (ward.D2) with Euclidean distance, with the number of bins set to be the number of beads/12 (Additional file 1: Fig. S13e). The expression score of each bin was calculated as the average of the relative gene expression intensities of beads assigned to that bin. Spatial coordinates of each bin were set to be the average x- and y-coordinates of its constituent beads. Clusters and type (malignant or non-malignant) of each bin were set to be the mode of those of their assigned beads. Lastly, the relative expression intensities in each bin were scaled by the number of UMIs per bin and expression values were capped at ≤ 1.4 and ≥ 0.6 to generate CNA scores.

### Clone determination

For clone determination, the total number of clusters, ${k}_{all}$, was determined to be the ${k}_{malignant}$ (the number of malignant clusters or putative clones) + 1 (one cluster assigned to the non-malignant population). To calculate ${k}_{malignant}$, malignant bins-only CNA scores (bins × genes) were hierarchically clustered by Ward’s hierarchical agglomerative clustering method [24] (ward.D2) with Euclidean distance with the cluster package v2.1.1 [25] in R and the number of malignant clusters was selected by testing ${k}_{malignant}$ = 2 to 10 clusters and choosing the ${k}_{malignant}$ that maximized the Silhouette score, ${k}_{max}$. Then, both malignant and non-malignant beads were hierarchically clustered by Ward’s hierarchical agglomerative clustering method [24] (ward.D2) with Euclidean distance with the cluster package v2.1.1 [25] using ${k}_{all}$ to cut the tree into discrete clusters.

### Differential gene expression analysis between CNA clusters

Differentially expressed genes were identified between a given CNA bead cluster and all other clusters (*p*_adj < 0.05) by a negative binomial generalized linear model [18] with the Bonferroni correction as implemented in Seurat v4, and ordered by decreasing average log_2_ fold change. Genes were tested for enrichment in Gene Ontology 2018 Biological Processes using Enrichr [26, 27].

### In silico Slide-seq simulated data

To generate an in silico Slide-seq dataset, cell/nucleus profiles were annotated for cell types and assigned to pseudo-spatial coordinates, followed by Gaussian smoothing of expression values to obtain simulated Slide-seq beads.

SnRNA-seq profiles for HTAPP-895-SMP-7359 and HTAPP-944-SMP-7479 were annotated as non-malignant or malignant based on SingleR v1.8.0 annotations (as described above) [19]. ScRNA-seq profiles for CID4530N and CID4535 were annotated as malignant if they belonged to the cancer epithelial or normal epithelial cell types (to capture mis-annotations or pre-malignant states) and non-malignant otherwise. A 3000 × 3000 square was created in silico to mimic the 3000 µm diameter of a Slide-seq puck. For each combined dataset (HTAPP MBC snRNA-seq dataset and primary breast cancer scRNA-seq dataset), two in silico spatial datasets were generated with either: (1) non-malignant cells/nuclei spatially separate from malignant cells/nuclei (“separated”) or (2) non-malignant cells/nuclei mixed with malignant populations (“mixed”). In each scenario, 6000 non-malignant cell/nucleus profiles were sampled (with replacement) from the two sc/snRNA-seq datasets combined (to provide distinct clones) and placed either at the top of the square at coordinate region [1:3000, 2701:3000] (“separated”) or across the square at coordinate region [1:3000, 1:3000] (“mixed”). Next, 7000 malignant cells/nuclei were randomly sampled (with replacement) from each sample. For the “separated” scenario, malignant cell/nucleus profiles were placed at the bottom of the square at coordinate region [1:3000, 1:2700]; for the “mixed” scenario, malignant cell/nucleus profiles were placed across the coordinate region [1:3000, 1:3000]. To test for recovery of clonal CNA profiles, malignant cell/nucleus profiles were titrated from each sample to create a rightward gradient of malignant sample 1 profiles with decreasing frequency and a leftward gradient of malignant sample 2 profiles with decreasing frequency. These gradients were established by dividing the square into 100 zones of horizontal width = 30 so that zones had sample 1:sample 2 ratios in 1% decrements (99:0, 98:1, … 0:99), creating a square containing 20,000 cells/nuclei (6000 non-malignant, 7000 malignant sample 1, and 7000 malignant sample 2) across a 3000 × 3000 region with gradient mixture of the two malignant cell/nucleus populations and either a separated or mixed non-malignant population.

Beads were simulated from the spatially positioned sc/snRNA-seq profiles by spatial Gaussian smoothing across the 3000 × 3000 square, with a relative Gaussian kernel width = $1500\cdot \sqrt{\frac{1}{\text{20,000}}}$ for 10,000 beads with randomly sampled, discrete x and y coordinates within the square and using actual bead size 10 µm [3] and cell size $1500\cdot \sqrt{\frac{1}{\text{20,000}}}$ estimates. This created a set of 10,000 beads with two-dimensional spatial coordinates and combined expression values of constituent cells/nuclei. The resulting in silico bead × gene expression matrix was used as input to SlideCNA and InferCNV for validation.

### Sensitivity analysis by down-sampling

For sensitivity analysis, reads from cell/nucleus profiles used for simulation were downsampled to 25%, 10%, and 5% (primary breast cancer scRNA-seq dataset) or 2% (HTAPP MBC snRNA-seq dataset) of their original counts across the entire dataset with the R package scuttle [28]. Note that 10% downsampling led to a median of 867.5 and 647 (HTAPP MBC dataset) and 530 and 387 (primary breast cancer scRNA-seq dataset) reads per bead for the “separated” and “mixed” simulations, respectively, which is comparable to the median bead read count for real Slide-seq data of 842 (HTAPP-895-SMP-7359) and 647 (HTAPP-944-SMP-7479).

### Spearman’s rank correlation of CNA scores

To compare CNA scores across pairs of methods (SlideCNA, InferCNV, CopyKAT, ABSOLUTE) and modalities (Slide-seq, snRNA-seq, WES), average CNA score per chromosomal arm was determined for each method-modality combination. In addition, the average allelic copy ratio per chromosomal arm for ABSOLUTE and WES was normalized by copy segment length and centered to be copy neutral at 1. Spearman’s rank correlation was calculated for each method-modality combination by average CNA scores per chromosomal arm. Chromosomal arms without CNA signal in some or all method-modality pairs were included for plots and excluded from Spearman’s rank correlation.

### Bead decomposition

Slide-seq data annotated by RCTD for HTAPP-895-SMP-7359 and HTAPP-944-SMP-7479 were processed by TACCO [13]. Reference expression profiles were generated using construct_reference_profiles and beads were decomposed using split_observations with a minimum of 10 counts. The resulting anndata object of decomposed beads was inputted into SlideCNA.

### Whole exome sequencing

DNA extraction, library construction, cluster amplification, and sequencing were performed as previously described [29].

Exome sequence data were processed using the Firecloud and Terra platforms (https://portal.firecloud.org/). A BAM file was produced with the Picard pipeline (http://broadinstitute.github.io/picard/) by aligning Illumina sequencing reads to the hg19 human genome build. Quality control modules within Firecloud/Firehose were applied to all sequencing data for comparison of the origin for tumor and normal genotypes and to assess fingerprinting concordance. Cross-contamination of samples was estimated using ContEst [30].

### Detection of CNAs in WES data

To properly compare somatic alterations in tumor samples, mutations were used as input for ABSOLUTE, which uses mutation-specific variant allele fractions (VAF) together with the computed purity, ploidy, and segment-specific allelic copy-ratio to compute cancer cell fractions (CCFs) and “absolute” copy number estimates [12]. MuTect was applied to identify somatic single nucleotide variants [31], insertions and deletions, and computational artifacts introduced by DNA oxidation [32] and formalin-fixed, paraffin-embedded sample preparation [33]. Somatic variants were further filtered as previously described [29]. To infer somatic copy number (copy-ratio segments labeled as an amplification, deletion, or copy number neutral), the WES data were segmented through the circular binary segmentation algorithm [31]. Somatic copy number was called using the calling copy-ratio functionality of the GATK [32]. To infer allele-specific copy ratios, all germline heterozygous sites in the germline non-malignant sample were mapped using GATK Haplotype Caller [33]. Read counts at germline heterozygous sites were then compiled to determine the copy profile of each homologous chromosome. The allele-specific copy profiles were segmented to produce allele-specific copy ratios.

### InferCNV

InferCNV v.1.18.1 was applied to RCTD-annotated Slide-seq data and snRNA-seq data after filtering for beads/nuclei with > 200 genes detected per bead/cell for HTAPP-878-SMP-7149, HTAPP-880-SMP-7179, HTAPP-895-SMP-7359, and HTAPP-944-SMP-7479 using the following parameter settings: cutoff = 0.1, denoise = T, HMM = T [7].

### CopyKAT

CopyKAT v1.1.0 was applied to RCTD-annotated Slide-seq data (min.genes = 100, ngene.chr = 1, KS.cuts = 0.1, and win.size = 20) and snRNA-seq data (min.genes = 200, ngene.chr = 5, KS.cuts = 0.1, and win.size = 30) for samples for HTAPP-878-SMP-7149, HTAPP-880-SMP-7179, HTAPP-895-SMP-7359, and HTAPP-944-SMP-7479 [9].

## Supplementary Information

Additional file 1: Supplementary Figures S1–S14.

Additional file 2: Review history.

## Data Availability

SlideCNA (R package) is published on CRAN (https://cran.r-project.org/web/packages/SlideCNA/) [34], available through GitHub under the MIT license with analysis vignettes: https://github.com/dkzhang777/SlideCNA [35] and https://github.com/dkzhang777/SlideCNA_Analysis [36], respectively, and is deposited on Zenodo alongside analysis vignettes as zipped GitHub repositories under the Creative Commons Attribution 4.0 International License (10.5281/zenodo.10658096) [37]. MBC Slide-seq, snRNA-seq, and WES data [38] are available as part of the HTAN-HTAPP data release through the HTAN portal (https://htan-portal-nextjs-git-fork-leexgh-mbc-publication-htan.vercel.app/publications/htapp_mbc_klughammer_2023?tab=overview) [39]. Raw sequencing data are available on dbGAP (accession number: phs002371, https://www.ncbi.nlm.nih.gov/projects/gap/cgi-bin/study.cgi?study_id=phs002371.v6.p1) [38], and linked through the HTAN data portal. In the HTAN database, HTAPP-878-SMP-7149 is HTA1_878_7149, HTAPP-880-SMP-7179 is HTA1_880_7179, HTAPP-895-SMP-7359 is HTA1_895_7359, and HTAPP-944-SMP-7479 is HTA1_944_7479. Additionally, MBC Slide-seq and snRNA-seq data are available on CELLxGENE, where samples can also be directly browsed and downloaded using the following link and selecting the respective samples: https://cellxgene.cziscience.com/collections/a96133de-e951-4e2d-ace6-59db8b3bfb1d. Primary breast cancer scRNA-seq data [11] are available at https://singlecell.broadinstitute.org/single_cell/study/SCP1039 [40]. Raw sequencing data are available on EGA (accession number: EGAS00001005173) [41]. Finally, the source code and a test dataset as input to the Jupyter notebook vignettes based on HTAPP-895-SMP-7359 has been deposited on Zenodo (10.5281/zenodo.10658096) [37] under the Creative Commons Attribution 4.0 International License.
