# Supplementary material for: SlideCNA: spatial copy number alteration detection from Slide-seq-like spatial transcriptomics data
Source: Genome Biol. 2025 May 2;26:112. doi: 10.1186/s13059-025-03573-y (PMC12046676; doi:10.1186/s13059-025-03573-y)
Supplement: Supplementary file 1 — Additional file 1: Supplementary Figures S1–S14. [file 13059_2025_3573_MOESM1_ESM.pdf]

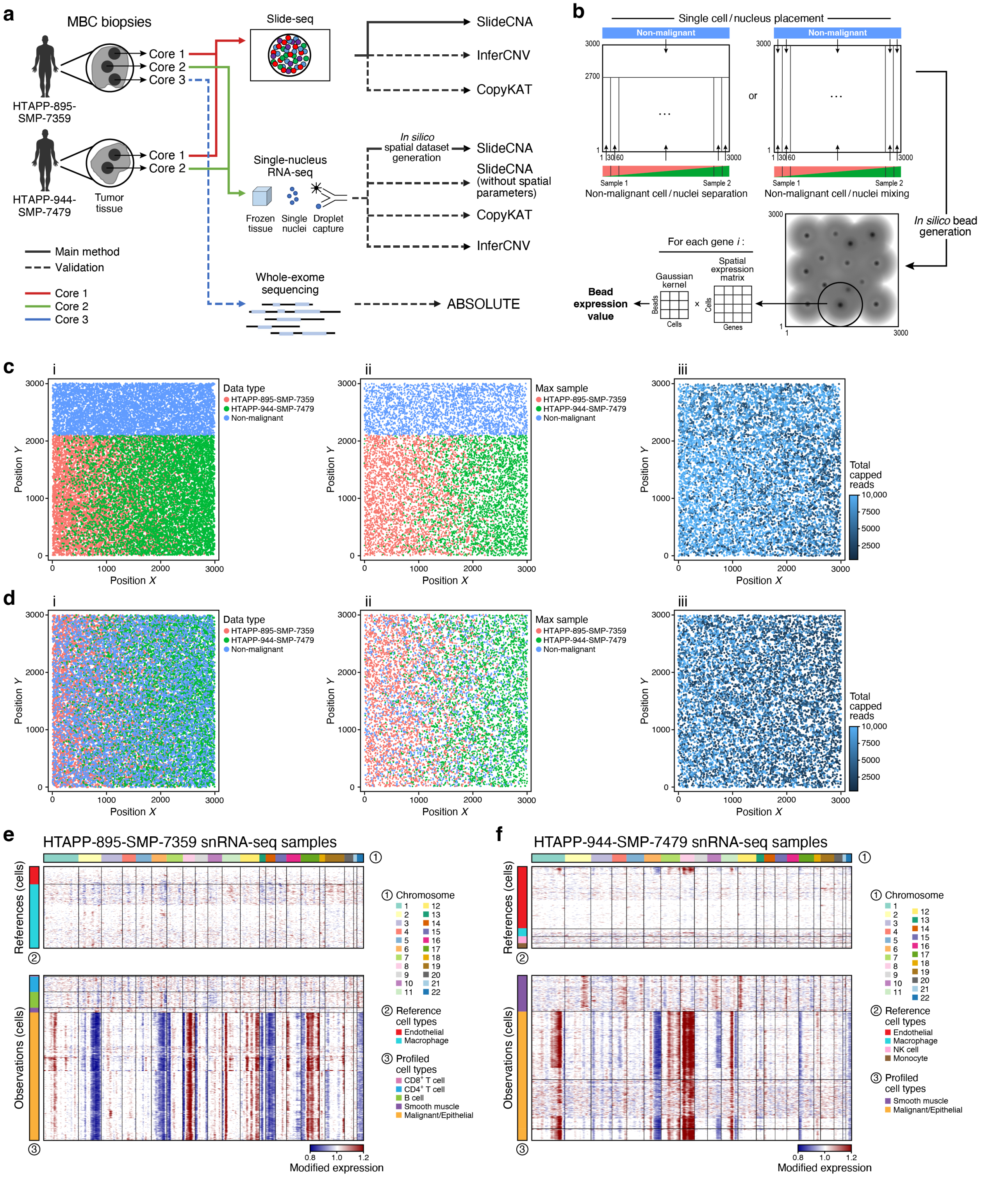

Fig. S1. SlideCNA analysis, validation, and benchmarking using MBC data. **a,** Schematic illustrating MBC data collection, Slide-seq and *in silico* spatial CNA analysis with SlideCNA, validation with orthogonal data (snRNA-seq and WES data), and benchmarking against alternative methods (InferCNV, CopyKAT). **b**, Schematic of generating the *in silico* datasets by placing snRNA-seq nuclei from each sample on a 3000x3000 square with either non-malignant nuclei separation or mixing and applying a Gaussian kernel to create *in silico* beads. **c,d,** i. Spatial plots of snRNA-seq nuclei; ii. the sample contributing the most reads to each *in silico* bead, and iii. the number of reads (capped at 10,000) per *in silico*-generated bead without downsampling for the *in silico* datasets with non-malignant separation **(c)** and non-malignant mixing **(d)**. **e,f**, InferCNV heat maps of HTAPP-895-SMP-7359 **(e)** and HTAPP-944-SMP-7479 **(f)** snRNA-seq samples.

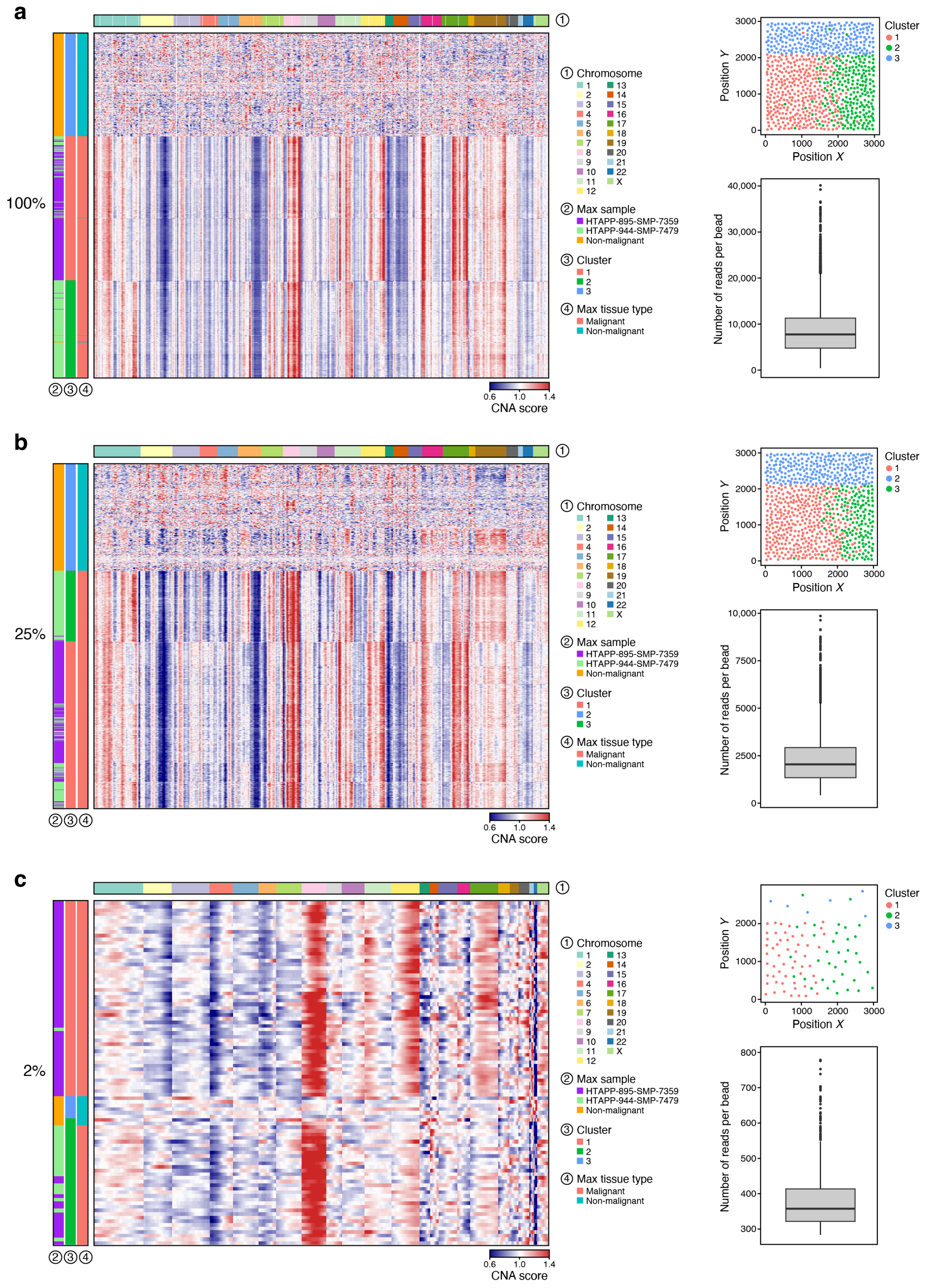

Fig. S2. *In silico* MBC snRNA-seq-derived spatial dataset with non-malignant separation results for downsampled counts. **a-c,** SlideCNA heat map (amplification > 1, deletion < 1), spatial plot of bins colored by assigned cluster, and boxplot of number of reads per bin after filtering for beads with >300 counts across all genes for the *in silico* spatial dataset with non-malignant separation with varying degrees of downsampling: without downsampling **(a)**, downsampled to 25% **(b)**, and downsampled to 2% **(c)**.

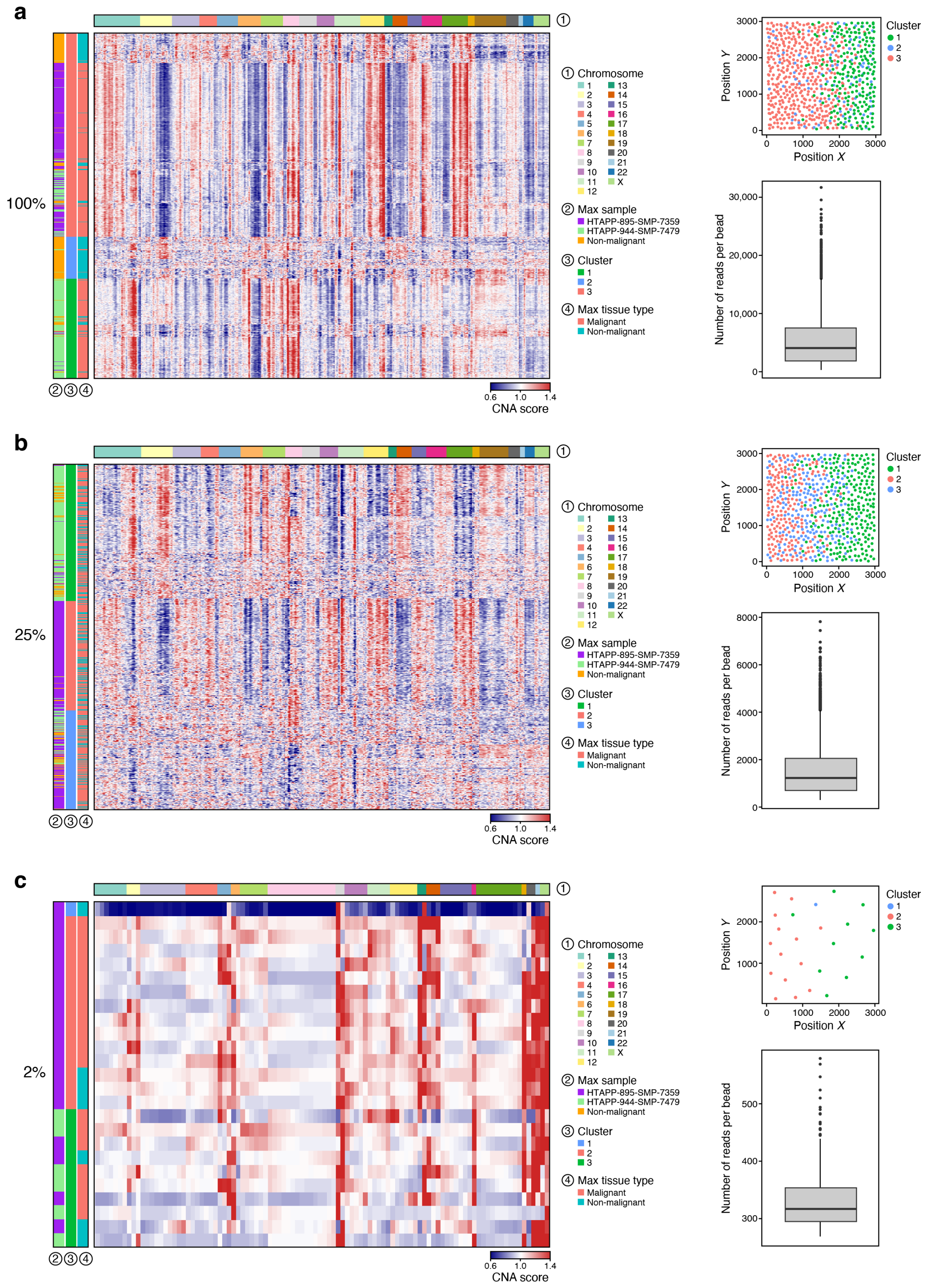

Fig. S3. *In silico* MBC snRNA-seq-derived spatial dataset with non-malignant mixing results for downsampled counts. **a-c,** SlideCNA heat map (amplification > 1, deletion < 1), spatial plot of bins colored by assigned cluster, and boxplot of number of reads per bin after filtering for beads with >300 counts across all genes for the *in silico* spatial dataset with non-malignant mixing and counts with varying degrees of downsampling: without downsampling (**a**), downsampled to 25% **(b)**, and downsampled to 2% **(c)**.

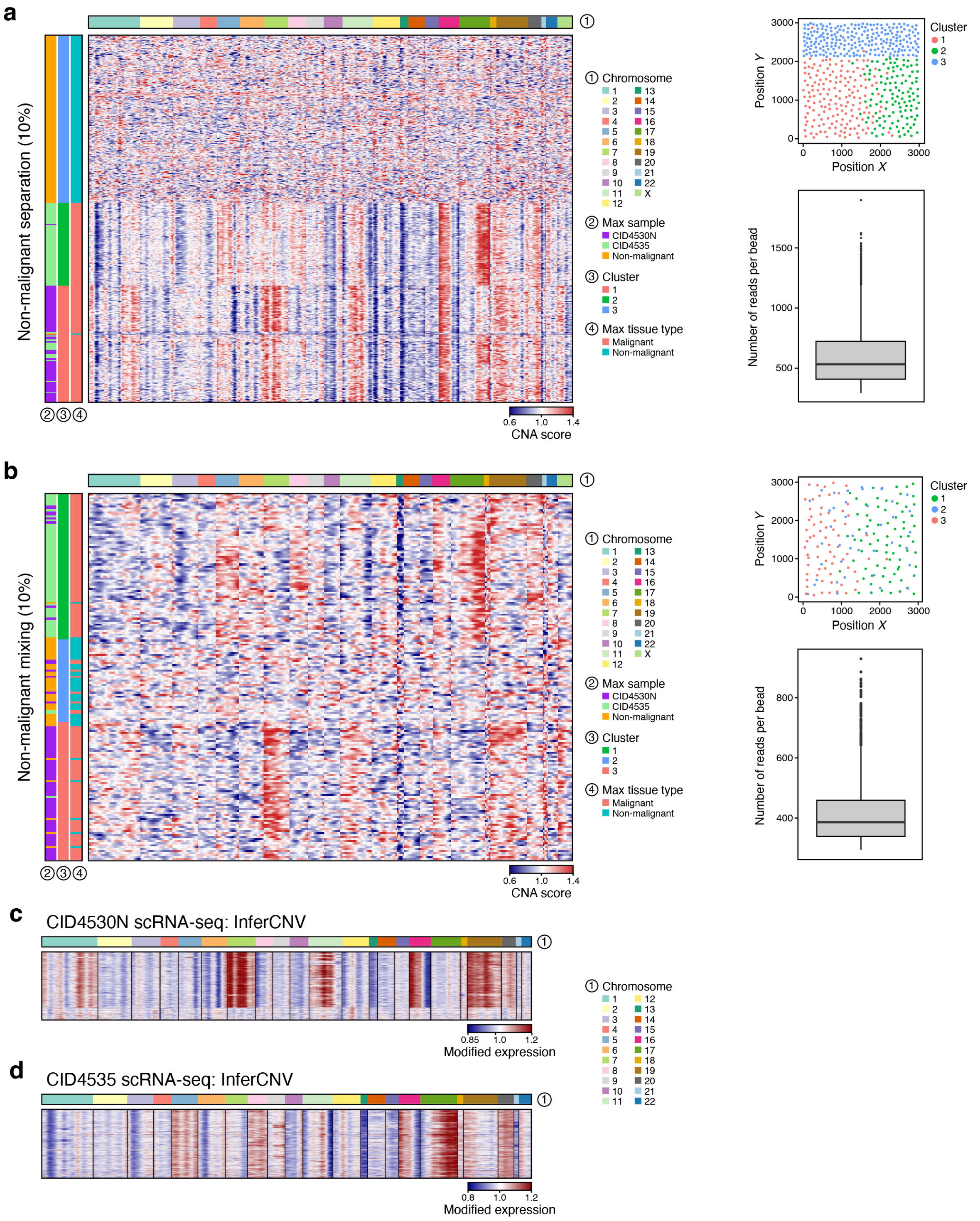

Fig. S4. *In silico* primary breast cancer scRNA-seq-derived spatial dataset results. **a,b,** SlideCNA heat map (amplification > 1, deletion < 1), spatial plot of bins colored by assigned cluster, and boxplot of number of reads per bin after filtering for beads with >300 counts across all genes for the *in silico* non-malignant-separated dataset **(a)** and non-malignant-mixed dataset **(b)** with counts downsampled to 10%. **c,d,** Summary of the InferCNV CNA profiles of malignant cells for scRNA-seq data of CID4530N **(c)** and CID4535 **(d).**

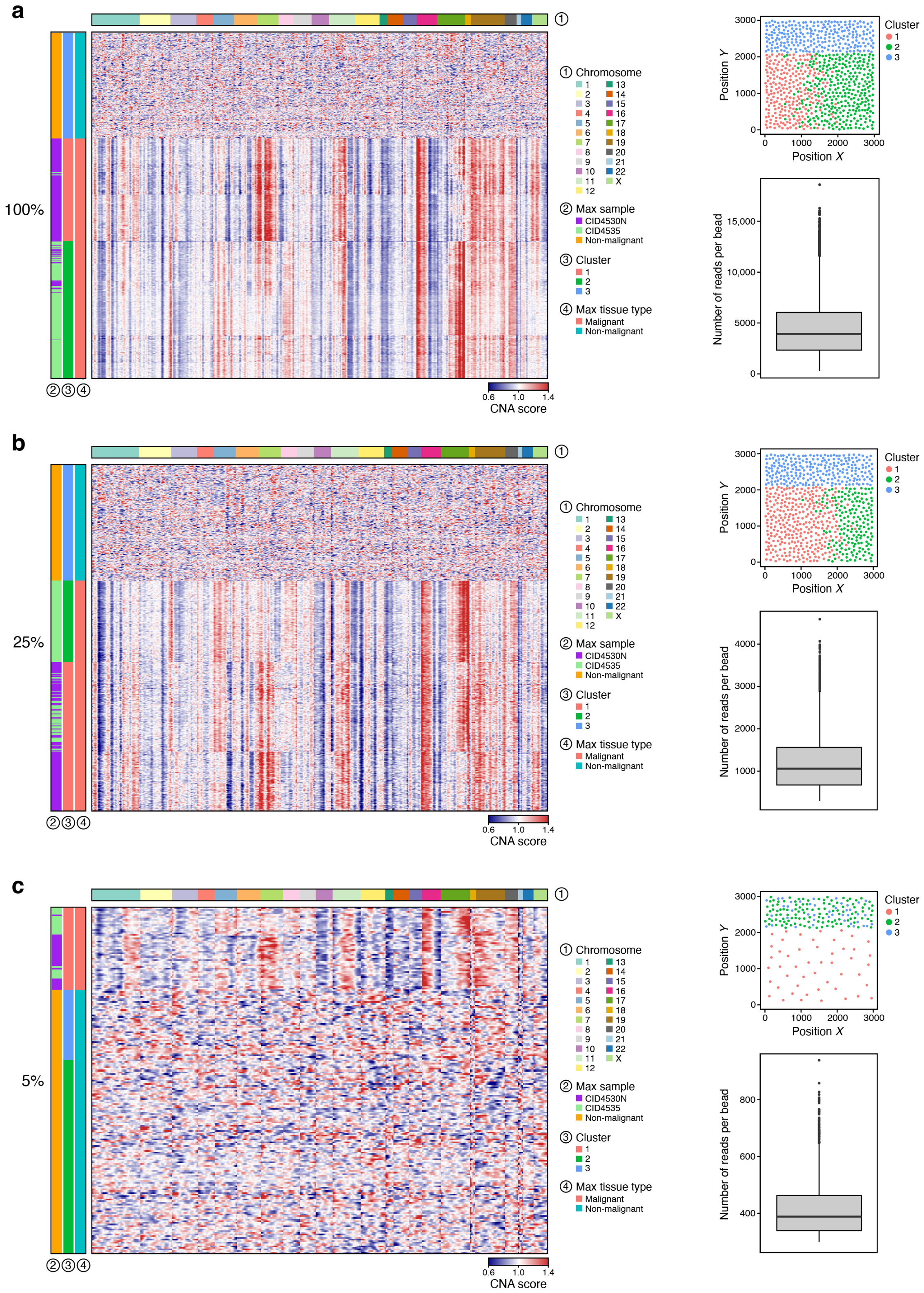

Fig. S5. *In silico* primary breast cancer scRNA-seq-derived spatial dataset with non-malignant separation results for downsampled counts. **a-c,** CNA heat map, spatial plot of bins colored by assigned cluster, and boxplot of number of reads per bin after filtering for beads with >300 counts across all genes for the *in silico* spatial dataset with non-malignant separation with varying degrees of downsampling: without downsampling **(a)**, downsampled to 25% **(b)**, and downsampled to 2% **(c)**.

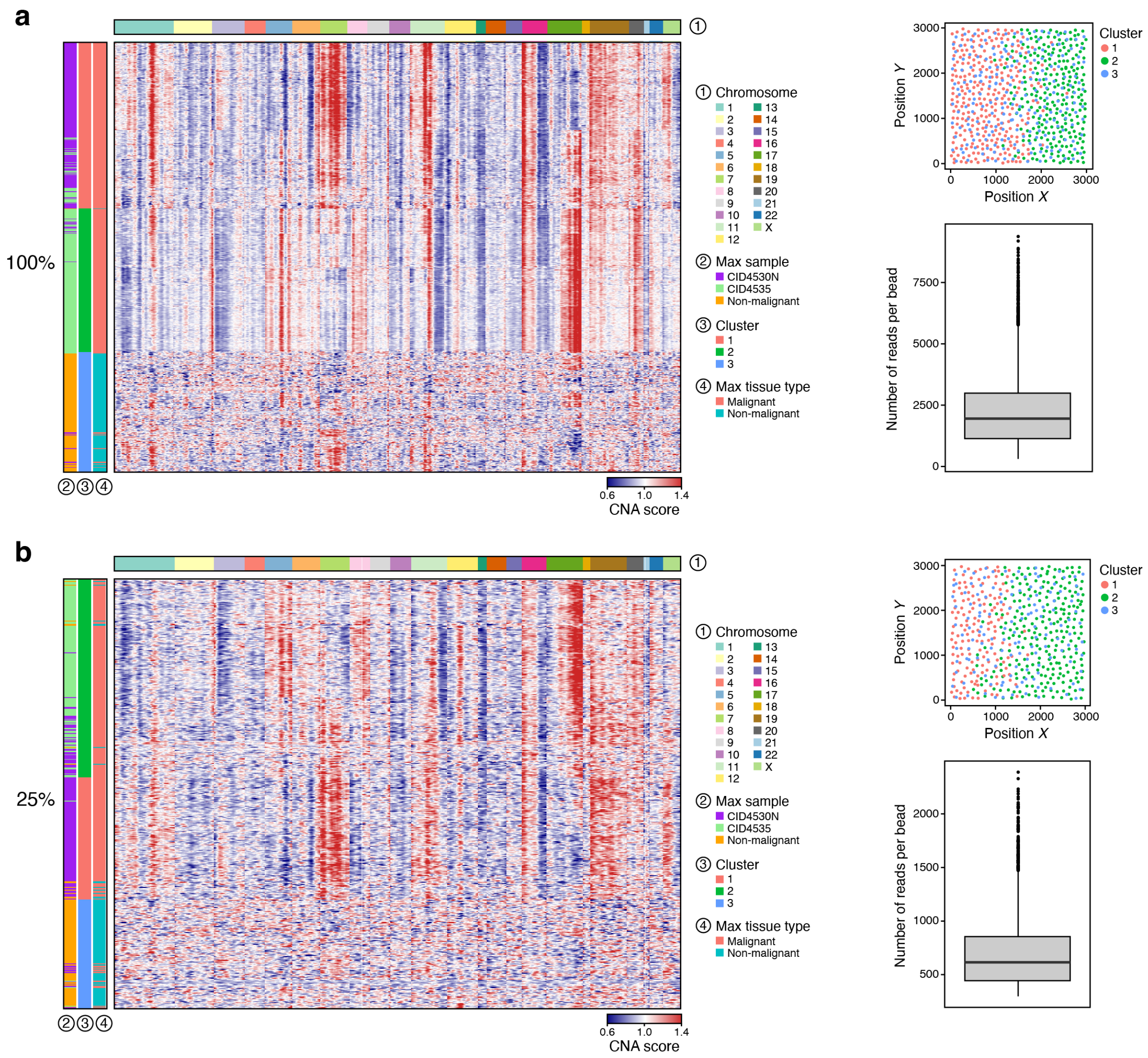

Fig. S6. *In silico* primary breast cancer scRNA-seq-derived spatial dataset with non-malignant mixing results for downsampled counts. **a-b,** CNA heat map, spatial plot of bins colored by assigned cluster, and boxplot of number of reads per bin after filtering for beads with >300 counts across all genes for the *in silico* spatial dataset with non-malignant mixing and counts with varying degrees of downsampling: without downsampling **(a)** and downsampled to 25% **(b)**. There were not sufficient malignant reads for downsampling to 5%.

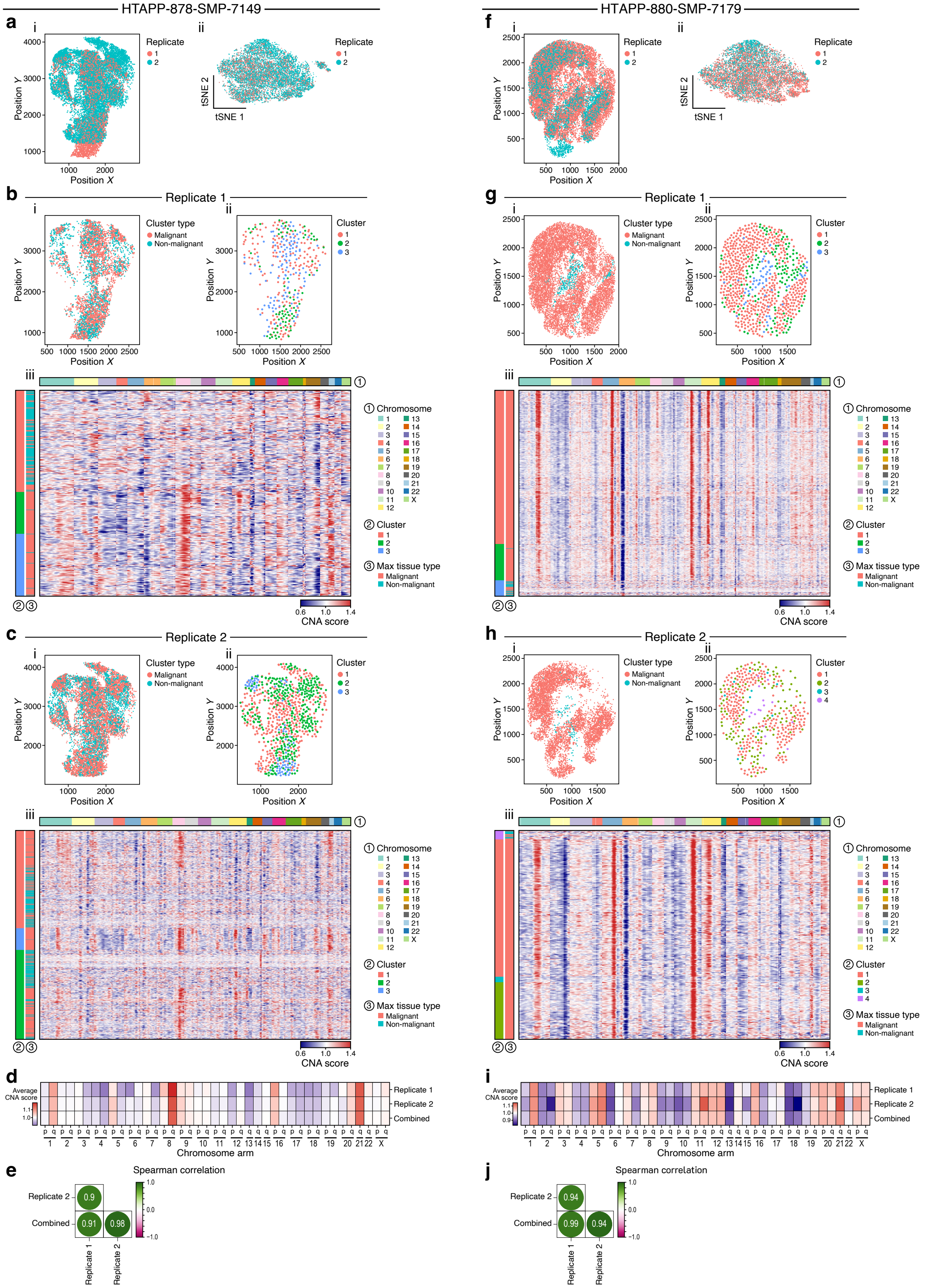

HTAPP-878-SMP-7149
HTAPP-880-SMP-7179
a
f
i
ii
Replicate
1
2
Position Y
Position X
tSNE 1
tSNE 2
b
g
Replicate 1
Cluster type
Malignant
Non-malignant
Cluster
3
4
iii
Chromosome
Cluster
Max tissue type
CNA score
0.6
1.0
1.4
c
h
Replicate 2
d
i
Average CNA score
Combined
Chromosome arm
e
j
Spearman correlation
0.9
0.91
0.98
0.94
0.99
0.94
Combined

Fig. S7. SlideCNA CNA patterns are consistent in Slide-seq data from HTAPP MBC across replicates. **a,f,** i. Combined spatial plot of two replicates of MBC Slide-seq data; ii. Combined TSNE plot of two replicates of MBC Slide-seq data. **b,c,g,h,** i. Spatial plot of Slide-seq beads annotated as non-malignant (blue) or malignant (pink); ii. Spatial plot of binned beads colored by SlideCNA-defined cluster designation; iii. SlideCNA heat map of malignant and non-malignant binned beads annotated with cluster assignment for Replicate 1 **(b,g)** and Replicate 2 **(c,h)**. **d,i,** Comparison of average SlideCNA CNA scores per chromosome arm for Slide-seq data of each replicate and both replicates combined for HTAPP-878-SMP-7149 **(d)** and HTAPP-880-SMP-7179 **(i)**. **e,j,** Pairwise Spearman correlation of CNA profiles as in panels **(d)** and **(i)** for HTAPP-878-SMP-7149 **(e)** and HTAPP-880-SMP-7179 **(j)**. **a-e** refer to sample HTAPP-878-SMP-7149 and **f-j** refer to sample HTAPP-880-SMP-7179.

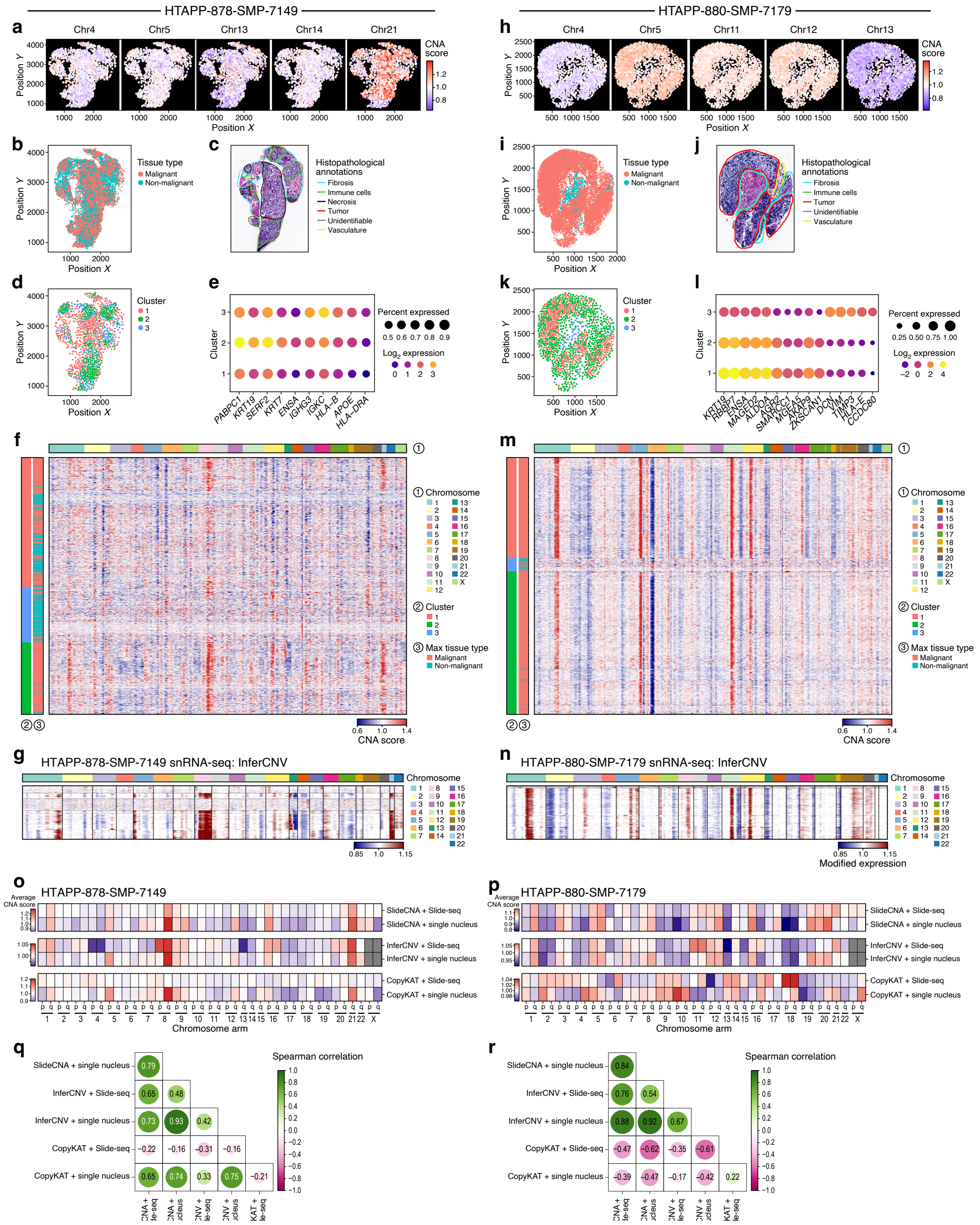

HTAPP-878-SMP-7149
HTAPP-880-SMP-7179
a
Chr4
Chr5
Chr13
Chr14
Chr21
CNA score
Position Y
Position X
h
Chr4
Chr5
Chr11
Chr12
Chr13
b
Tissue type
Malignant
Non-malignant
c
Histopathological annotations
Fibrosis
Immune cells
Necrosis
Tumor
Unidentifiable
Vasculature
i
j
d
Cluster
e
Percent expressed
Log2 expression
PABPC1
KRT19
SERF2
KRT7
ENSA
IGHG3
IGKC
HLA-B
APOE
HLA-DRA
k
l
KRT19
RBBP7
ENSA
MAGED2
ALDOA
AGR2
SMARCC1
MGEA5
AKAP9
ZKSCAN1
DCN
VIM
TIMP3
HLA-E
CCDC80
f
m
① Chromosome
② Cluster
③ Max tissue type
CNA score
g
HTAPP-878-SMP-7149 snRNA-seq: InferCNV
n
HTAPP-880-SMP-7179 snRNA-seq: InferCNV
Chromosome
Modified expression
o
HTAPP-878-SMP-7149
p
HTAPP-880-SMP-7179
Average CNA score
SlideCNA + Slide-seq
SlideCNA + single nucleus
InferCNV + Slide-seq
InferCNV + single nucleus
CopyKAT + Slide-seq
CopyKAT + single nucleus
Chromosome arm
q
r
Spearman correlation

Fig. S8. SlideCNA identifies spatial CNA patterns in two Slide-seq MBC samples with spatially combined replicates from Fig. S7. **a-g** refer to sample HTAPP-878-SMP-7149 and **h-n** refer to sample HTAPP-880-SMP-717. **a,h**, Spatial plot of beads colored by mean CNA score across the indicated chromosomes, selected to demonstrate a range of spatial CNA patterns . **b,i,** Spatial plot of beads annotated as non-malignant (blue) or malignant (pink) with non-malignant beads serving as reference for SlideCNA. **c,j,** H&E stains of consecutive sections matching the Slide-seq samples with histopathological annotations. **d,k,** Spatial plot of binned beads colored by SlideCNA-defined cluster designation on spatial axes. **e,l,** Top DEGs for each cluster detected from the SlideCNA profile. DEGs were colored by average $\log_2$ cluster expression and sized by the percent of beads expressing that gene in the cluster (negative binomial generalized linear model p-adj < 0.05). **f,m,** SlideCNA heat map of malignant and non-malignant binned beads annotated with cluster assignment. **g,n,** Summary of the InferCNV CNA profiles of malignant nuclei for snRNA-seq data of HTAPP-878-SMP-7149 **(g)** and HTAPP-880-SMP-7179 **(n). o,p,** Comparison of average SlideCNA, InferCNV, and CopyKAT CNA scores per chromosome arm for both Slide-seq and snRNA-seq data for HTAPP-878-SMP-7149 **(o)** and HTAPP-880-SMP-7179 **(p)**. **q,r,** Pairwise Spearman correlation of CNA profiles as in panels **(o)** and **(p)** for HTAPP-878-SMP-7149 **(q)** and HTAPP-880-SMP-7179 **(r)**.

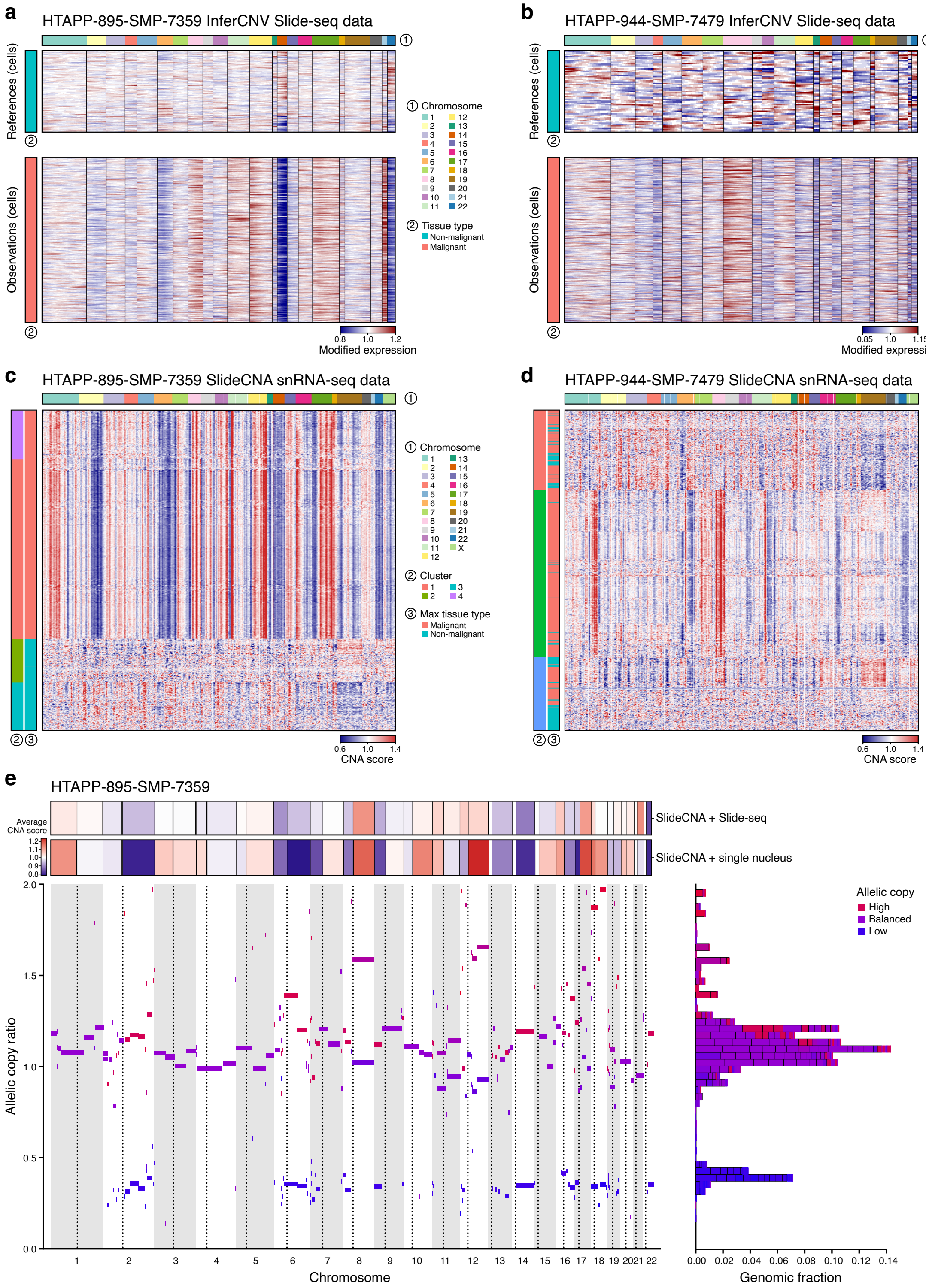

Fig. S9. Complementary CNA analysis for samples HTAPP-895-SMP-7359 and HTAPP-944-SMP-7479. **a,b,** InferCNV heat maps of HTAPP-895-SMP-7359 **(a)** and HTAPP-944-SMP-7479 **(b)** Slide-seq data, using the same reference beads as those used for SlideCNA. **c,d,** SlideCNA heat maps of HTAPP-895-SMP-7359 **(c)** and HTAPP-944-SMP-7479 **(d)** snRNA-seq data. **e,** ABSOLUTE CNA plot of the HTAPP-895-SMP-7359 WES data with average CNA scores per chromosome arm of the SlideCNA analyses on Slide-seq and snRNA-seq data (top).

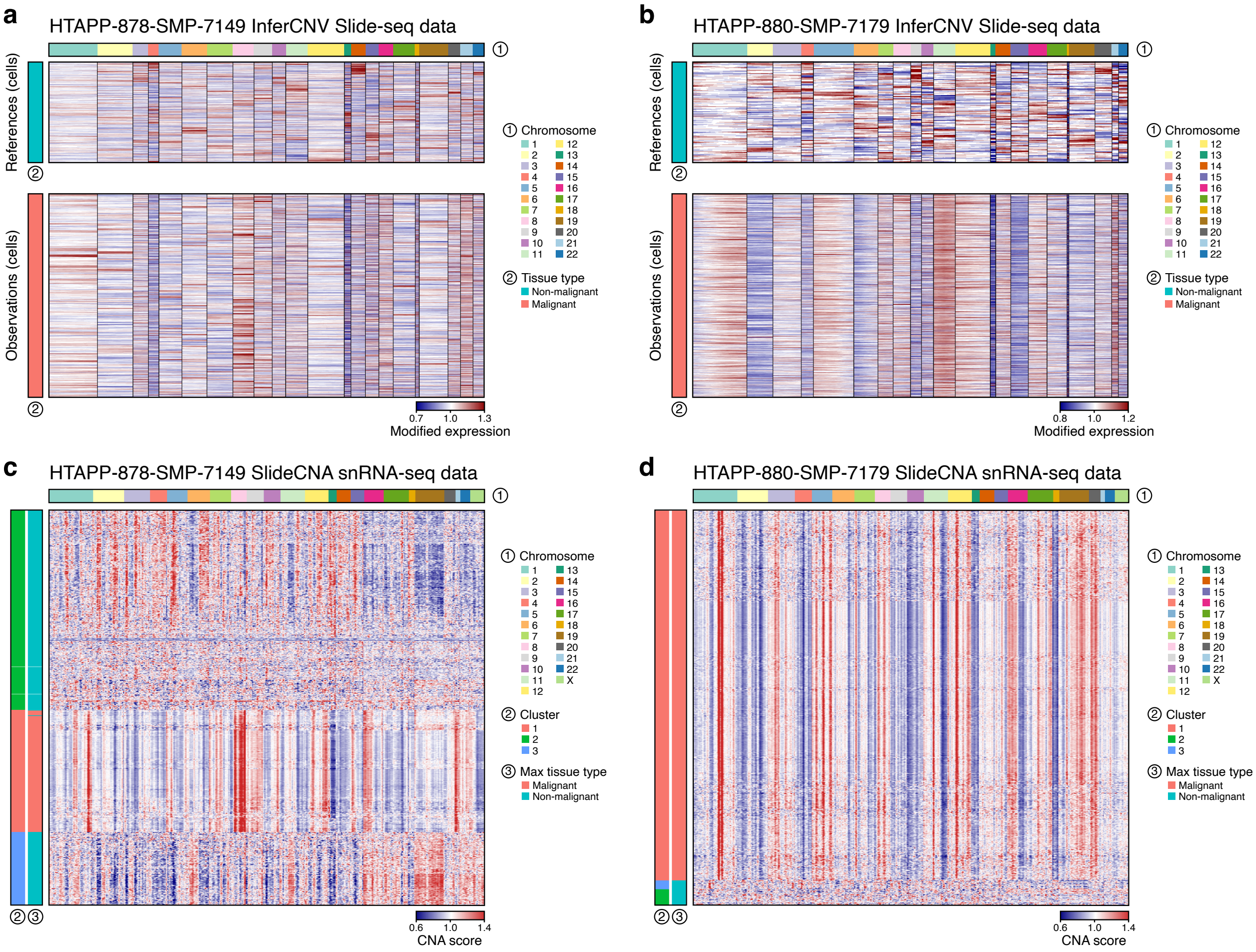

Fig. S10. Complementary CNA analysis for samples HTAPP-878-SMP-7149 and HTAPP-880-SMP-7179. **a,b,** InferCNV heat maps of HTAPP-878-SMP-7149 **(a)** and HTAPP-880-SMP-7179 **(b)** Slide-seq data, using the same reference beads as those used for SlideCNA. **c,d,** SlideCNA heat maps of HTAPP-878-SMP-7149 **(c)** and HTAPP-880-SMP-7179 **(d)** snRNA-seq data.

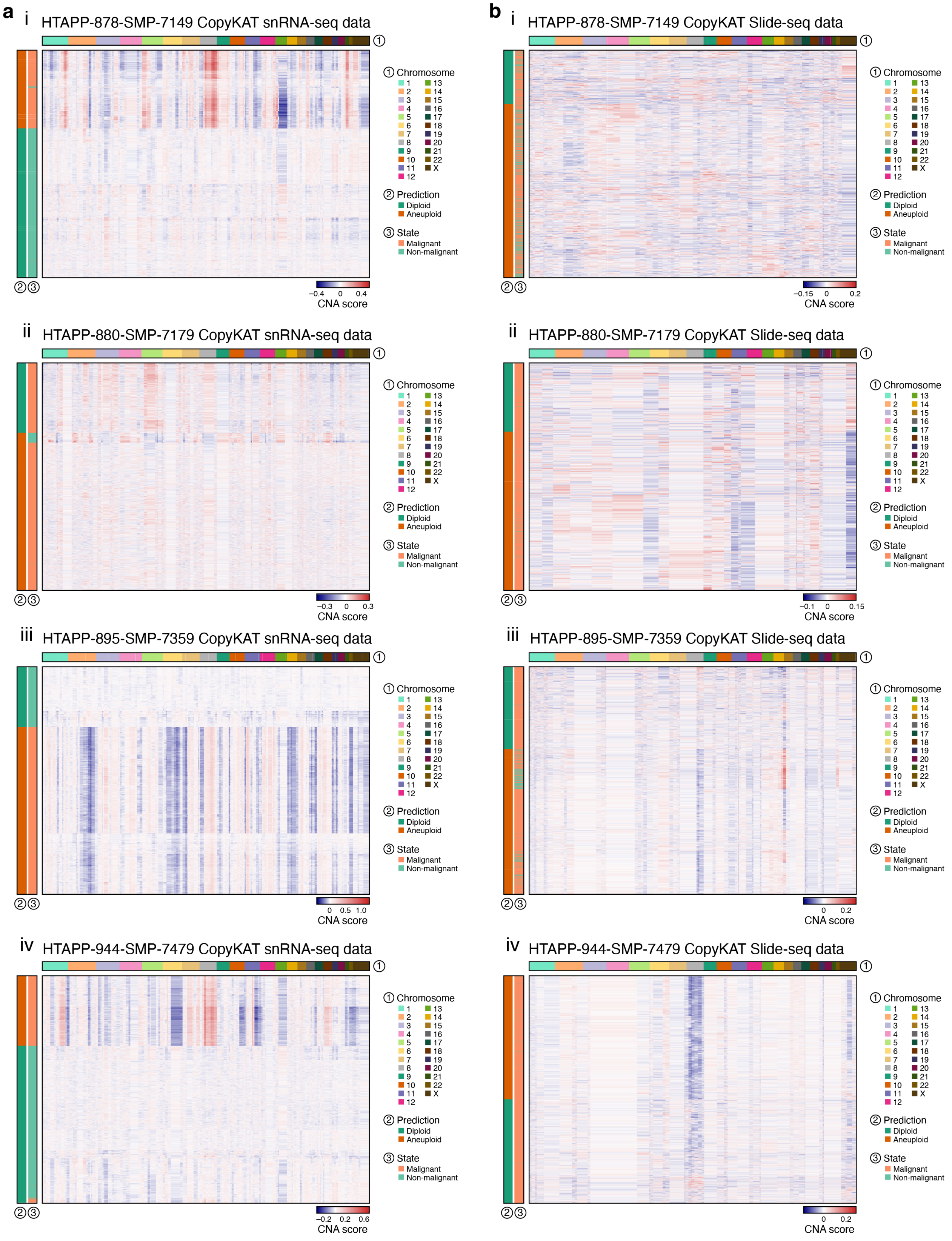

Fig. S11. Comparison of CNA patterns with CopyKAT for snRNA-seq and Slide-seq data. **a,b,** CopyKAT heat map of CNA scores and predicted ploidy for snRNA-seq data **(a)** and Slide-seq data **(b)** for HTAPP-878-SMP-7149 (i), HTAPP-880-SMP-7179 (ii), HTAPP-895-SMP-7359 (iii), and HTAPP-944-SMP-7479 (iv).

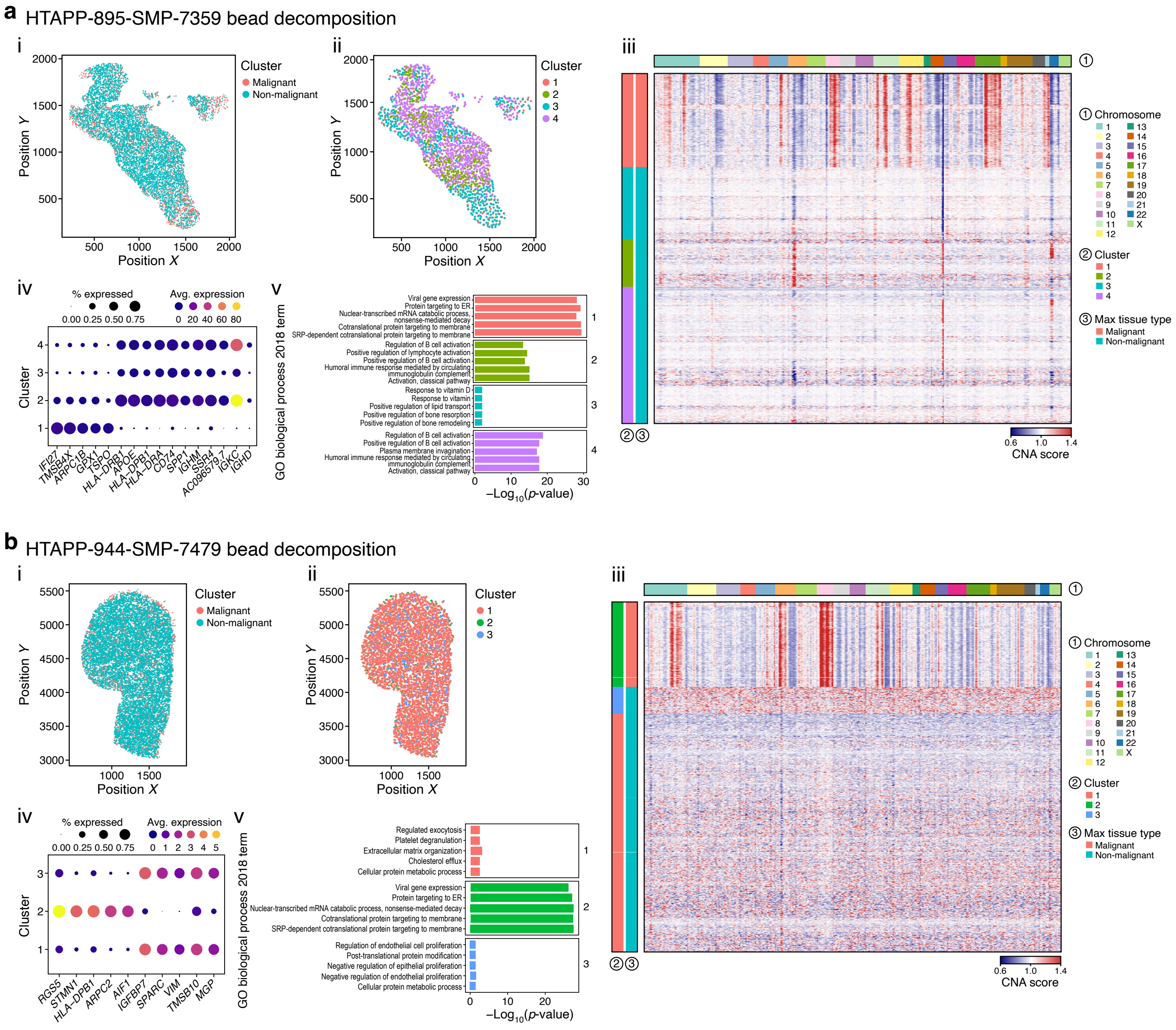

Fig. S12. SlideCNA captures spatial CNA patterns in Slide-seq data after bead decomposition by cell type. **a,b,** i. Spatial plot of decomposed beads annotated as non-malignant (blue) or malignant (pink). ii. Spatial plot of binned, decomposed beads colored by SlideCNA-defined cluster designation. iii. SlideCNA heat map of malignant and non-malignant binned decomposed beads annotated with cluster assignment. **(a)** refers to sample HTAPP-895-SMP-7359 and **(b)** refers to sample HTAPP-944-SMP-7479.

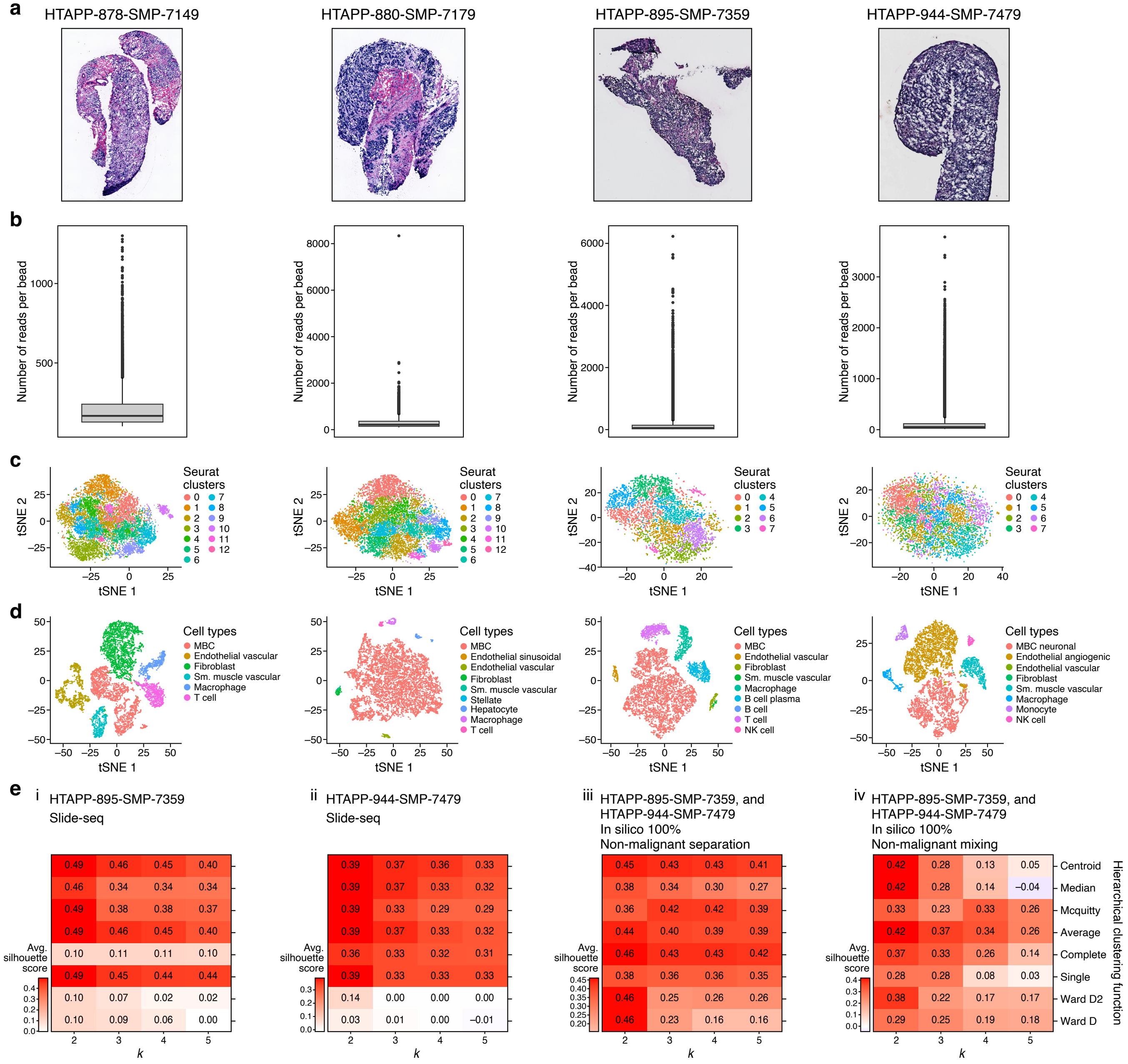

Fig. S13. Supplemental information for the MBC Slide-seq and snRNA-seq data. **a-d,** H&E stains of adjacent sections of the Slide-seq samples **(a),** boxplots of number of reads per bead of the Slide-seq data **(b),** t-SNE plots of Slide-seq data Seurat clusters **(c)**, and t-SNE plots of snRNA-seq data cell types **(d)** for HTAPP-878-SMP-7149, HTAPP-880-SMP-7179, HTAPP-895-SMP-7359, and HTAPP-944-SMP-7479. **e,** Silhouette scores for each hierarchical clustering method applied to determine the number of clusters, *k*, of malignant binned beads across *k* = 2 to 5 in the HTAPP-895-SMP-7359 Slide-seq sample (i), HTAPP-944-SMP-7479 Slide-seq sample (ii), MBC *in silico* dataset with non-malignant separation and no downsampling (iii), and MBC *in silico* dataset with non-malignant mixing and no downsampling (iv).

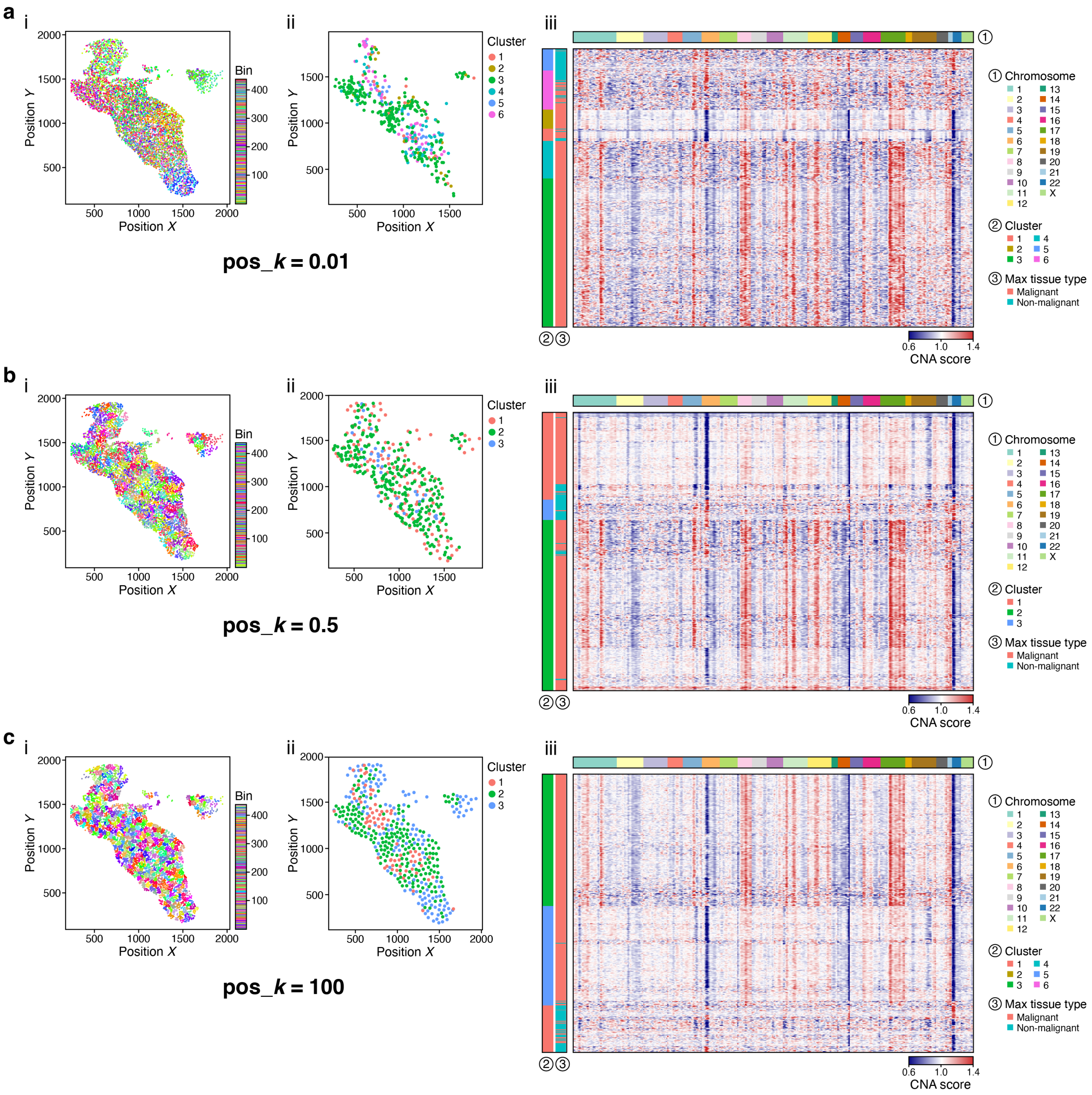

Fig. S14. Bootstrapping the SlideCNA spatial binning parameter for the HTAPP-895-SMP-7359 sample. **a-c,** i. Spatial plot of Slide-seq beads colored by SlideCNA-defined bin assignments. ii. Spatial plot of binned beads colored by SlideCNA-defined cluster designation. iii. SlideCNA heat map of malignant and non-malignant binned beads annotated with cluster assignment. SlideCNA was run with values of *pos_k* = 0.01 **(a)**, 0.5 **(b)**, and 100 **(c)** (default: 55) with a higher *pos_k* indicating a greater weight assigned for spatial positioning in binning.
