# Supplementary material for: SlideCNA: spatial copy number alteration detection from Slide-seq-like spatial transcriptomics data
Source: Genome Biol. 2025 May 2;26:112. doi: 10.1186/s13059-025-03573-y (PMC12046676; doi:10.1186/s13059-025-03573-y)
Supplement: Supplementary file 2 — Additional file 2: Review history. [file 13059_2025_3573_MOESM2_ESM.docx]

Review History

**First round of review**

**Reviewer 1**

**Were you able to assess all statistics in the manuscript, including the appropriateness of statistical tests used?** Yes

**Were you able to directly test the methods?** See report

**Comments to author:**

Zhang et. al present their novel computational method (SlideCNA) which estimates copy-number alterations (CNA) based on RNA expression captured in a spatial manner utilizing Slide-seq technology.

The authors report on the proof-of-principle of SlideCNA for spatially-extended/simulated in silico snRNA-Seq data and Slide-Seq experiments from two distinct breast cancer liver metastases (MBC) in which potential subclones with distinct CNA-profiles could be observed within spatial dimensions.

This method extends the usability of spot-based sequencing (i.e., transfer of RNA from tissue sections onto an arrayed surface with predefined beads or barcodes for signal capturing with spatial information) with the ability to not only determine the presence and (estimated) abundance of RNA molecules but also to use this information to estimate spatial-aware CNA events in low sparsity data. This has an inherent difficulty as these spots typically capture ~10μm tissue sections containing a mixture of multiple cells (or fractions) rather than being single-cell resolution which could obfuscate signals.

To overcome this difficulty and under the assumption that spatially proximal and expression-similar beads are likely to have similar CNA, SlideCNA first determines clusters of similar spots (or beads) based on both their spatial distance and reference-smoothed bead-expression profiles. Briefly, gene-wise expressions are first normalized/centered against the mean expression within an a priori defined set of reference beads and thereafter used to designate smoothed expression bins (of k genes). To address the sparsity of Slide-Seq and potentially increase signal, (adjacent) beads are binned together mainly based on spatial distance and also on smoothened expression profiles; both combined and captures as pseudo-distances. The mean values of constituent beads within these bead-bins were used as representation for the x/y spatial coordinates and gene-expression per genomic bin (which are scaled by number of UMIs per bin and subsequently capped) are used to generate bead-bin CNA-scores. Next, CNA-scores of malignant-only bead-bins are clustered to determine potential subclones which thereby reflect both spatial and differences in genomic bin expressions.

This is an interesting approach as it directly extends the functionality of current Slide-Seq experiments and therefore warrants eventual publication.

However, this current manuscript should be improved by a more rigorous curation and detailed (automated/reproducible) methodology of the chosen metrics, samples, reference set of non-malignant beads/cells and assessment of chromosomal-arm levels used in the final comparison between SlideCNA/inferCNV on Slide-Seq and non-spatially aware snRNA-Seq vs. WES/ABSOLUTE. In addition, the code and workflow itself (as deposited on Github) currently lack example data, vignettes and unit tests which makes assessing the robustness and validity of the code impossible and non-reproducible.

My major and minor comments on the current version are the following:

Comment #1 - Annotation of non-malignant cells in Slide-Seq.

The current annotation of non-malignant cells is not 100% convincing.

Please elaborate on the manual malignant/non-malignant annotation of SNN-based Seurat clusters within the Slide-Seq data, e.g., HTAPP-895-SMP-7359 (line 11, page 34). Could this be replicated in an automated and/or reproducible manner?

In addition, it is unclear how the top malignant/non-malignant clusters were chosen for the 'malignant gene-set' analysis (page 11; line 46-47). E.g., which DEGs an GO terms (and direction thereof) were defined as 'malignant'?

Fig. 2d detailing the DEGs between the CNA-clusters in HTAPP-895-SMP-7359 (where most non-malignant beads seem to fall into binned bead CNA cluster 3) show only three DEGs between the non-malignant and malignant CNA clusters. This may possibly indicate that the manual designation of these non-malignant beads may need some additional curation as other beads have likely also captured non-malignant cells and therefore rendering a differential expression analysis difficult unless the expression of genes fluctuates heavily within the malignant clusters.

Furthermore, the authors transplanted these 'reference' non-malignant beads from HTAPP-895-SMP-7359 onto HTAPP-944-SMP-7479 (with spatial offset) as the latter sample lacked intrinsic 'non-malignant' beads (according to the manual annotation).

As these non-malignant cells are the baseline reference and thereby the foundation against which CNA-scores are determined with SlideCNA, I would have preferred a more thorough (and reproducible) investigation into the confidence of these 'non-malignant' cells.

Could the authors also add additional histopathological information based on H&E on these samples supporting their decision-making?

Adding DAPI or similar staining of the tissue section to Fig. 2 (rather than just bead signal in Fig. 2a) would also clarify tissue structures. It is currently rather unclear what the 'white space' is and to what the sparse outer lying beads signals refer to.

Comment #2 - Selection and importance of spatial and expression profile weights in calculating the pseudo-distance.

The authors should provide more detailed explanation of how tuning parameters are chosen.

The authors have used k=55 and k=1 for weighing the spatial and expression profiles respectively to calculate the pseudo-distance matrix on which subsequent bead-clustering is performed.

Currently, there is no rationale for these chosen values beyond selecting for spatially close beads over (divergent) expression profiles. Could the authors explain their chosen weights (k=55 and k=1) and the dependency of SlideCNA for various iterations of k (e.g., bootstrapping of CNA clusters with various employed weights) to give users a feeling for which weights would be preferable in their samples and how much this affects the robustness of CNA clusters.

Comment #3 - Transplantation of non-malignant cells between MBC samples.

The approach of cut and pasting beads to another samples raises questions as to whether this introduces biases from batch effects.

Fig 2i reveals more uniform expression differences (DEGs) between the transplanted beads vs. the host malignant beads (CNA cluster 1 and 2 vs. 3) compared to the CNA cluster comparison within the donor sample in which I had expected more (or similar) DEGs between the malignant/non-malignant cluster. This might indicate that there are truly no non-malignant cells in cluster 3 (original beads of host) and that a clean biological comparison has been performed, or that there are also other subtle (both non-uniform and uniform) batch-effects in place which cause an increase of DEGs such as sample-specific batch-effects.

The authors claim that the transplantation of these non-malignant cells between Slide-Seq samples is a viable approach (page 12; line 4-9) with only a reference to the GitHub repository of their previous methodology (inferCNV). This reviewer is unable to find the supporting evidence for this statement within this repository such as an inter-sample comparison between the expression profiles of non-malignant cells in multiple Slide-Seq (or other scRNA-Seq) experiments which altogether rule out the occurrence of sample-specific batch-effects.

Could the authors indicate where exactly within this repository such a comparison would be located and/or provide additional references or supplemental data supporting this claim; as sample-specific batch effects have long been a known factor in the analysis of sequencing data, including scRNA-Seq.

Comment #4 - Overall comparison of copy-number profiles between methodologies (inferCNV/SlideCNA) and technologies (Slide-Seq, snRNA-Seq and WES).

The authors should conduct a more rigorous comparison to copy number alteration data from an established technology.

The comparison of copynumber profiles as estimated by ABSOLUTE on whole-exome sequencing (WES) inferCNV/SlideCNA on the Slide-seq and the snRNA-seq (without spatial parameters) lacks a reproducible methodology. I.e., what was the schematic to 'manually' consider arms to be deleted or amplified (Fig 2i-k) from the data of (among others) Fig. S4 and how robust is this calling?

For instance, S4E might also indicate an allelic balance/CN-neutral category for 2p yet Fig. 2i has 2p listed Deleted' under the WES category (which corresponds with SlideCNA). How exactly to interpret bead-wise Slide-seq/snRNA-seq (Fig. S4a-d) CNA-scores for a general sample-wise arm-level estimate is even more prone to personal bias if manual annotation is performed.

Furthermore, was this calling performed in a blinded manner (e.g., WES results were kept from assignee(s) prior to calling arms in snRNA/Slide-seq) or could there be (albeit unknown) bias in manual calling?

For instance, could the authors perhaps use CNA-scores of similar genomic windows to estimate the (per arm) similarity between the compared approaches rather than visual inspection and annotation?

In addition, it is unclear how SlideCNA was exactly performed on the snRNA-seq "with non-spatial parameters" (page 6; line 11-13). Does this mean that only X_expression (genes x beads with k_expression =1) was given with an empty X_spatial with _kspatial=0 whilst otherwise implementing the same algorithm (page 12-14; SlideCNA) or was another implementation used?

Comment #5 - Assessment of code/workfow

The package (https://github.com/dkzhang777/SlideCNA/) is currently missing a vignette, example data and unit tests to determine the robustness of the code and functions itself. For instance, the package does not currently satisfy the minimal requirements to be incorporated within BioConductor which is a good benchmark to assess minimal reproducibility and robustness.

Could the authors add this to the package to allow for a review of the workflow/code and to assess the user-friendliness? For instance, the description on how the metadata should be given (md in run_slide_cna()) is currently only available on the README rather than described within the function with accompanying example data.

As such, this reviewer could currently not perform, reproduce nor assess the validity of the package and SlideCNA.

Minor comments

1. (Page 11; Line 14) Annotation of cell-types using SingleR.

Please specify the used reference (e.g., Human Primary Cell Atlas (Mabbott et al. 2013) used to annotate cell-types with SingleR.

2. De-novo reference bead selection in Slide-Seq (M&M).

The term "bead cluster" (i.e., SNN-based clusters from the Seurat workflow on the Slide-seq data) can be seen as confusing with the (later defined) pseudo-distance calculated bead clusters in the CNA analysis and would benefit from a distinct term to distinguish the two (e.g., SNN-derived Slide-Seq bead cluster).

3. Suppl. Fig S2, S3 and S6

Typo in y-axis title of boxplot; "ber bead" rather than "per bead".

4. X-ticks Suppl. Fig. 6c-d.

Why are there five x-axis tick-lines if it is a single group/boxplot?

5. Definition of non-malignant clonal cluster.

As hierarchical clustering on CNA-scores is only performed on the malignant bead-bins and all non-malignant bead-bins are defined as a separate category (rather than through clustering), it might be clearer to a reader to annotate this non-malignant group not as a separate cluster (with incremental/decreasing cluster ID/name) but simply as 'Non-malignant' or similar to emphasize that these were not defined as a group by clustering but through other annotation.

6. Missing M&M on STARCH

Please describe the usage, version and any non-default parameters used in the STARCH analysis within the Methods section. Currently, this is only present within the results (Page 7; line 11-29).

7. Please double-check the spelling within the SlideCNA code. E.g., the main function (run_slide_cna) has a typo within its required parameters (OUPUT_DIRECTORY).

8. Authors claim that their previous method (InferCNV) is the current gold standard for CNA-detection in scRNA-seq (page 6, line-26). Do the authors have additional support for this claim?

E.g., how does it compare with the current adoptions of the widely-used CopyKAT method for scRNA-Seq which showed closer approximation to bulk reference DNA copy number profile (at 220Kb genomic resolution) in their comparison to inferCNV? (Gao R, Bai S, Henderson YC, Lin Y, Schalck A, Yan Y, Kumar T, Hu M, Sei E, Davis A, Wang F, Shaitelman SF, Wang JR, Chen K, Moulder S, Lai SY, Navin NE. Delineating copy number and clonal substructure in human tumors from single-cell transcriptomes. Nat Biotechnol. 2021 May;39(5):599-608. doi: 10.1038/s41587-020-00795-2. Epub 2021 Jan 18. PMID: 33462507; PMCID: PMC8122019)

**Reviewer 2**

**Were you able to assess all statistics in the manuscript, including the appropriateness of statistical tests used?** Yes

**Were you able to directly test the methods?** No

Zhang et al. developed SlideCNA, a toolkit that infers copy number alteration (CNA) from slide-seq spatial transcriptomics data. Similar to inferCNV, SlideCNA uses contiguous chromosomal expression changes to infer copy number alterations. Thus, both tools were based on assumption that alteration of expression abundance was primary due to change in gene dosage. Using snRNA-seq of metastatic breast cancer tumor core biopsy from two patients, authors constructed an in-silico slide-seq data set to demonstrate the performance of SlideCNA. Finally, authors applied SlideCNA on the actual slide-seq on a separated core biopsies from same patients.
In general, it is a very challenging task to infer CNA with altered gene expression in cells. The accuracy and granularity achieved with these types of method were very limited (Slyper M., et al. Nature Medicine 26, 792-802 (2020)). SlideCNA does not offer any significant innovations and is primarily an improved version of inferCNV. Nevertheless, it would be helpful to understand the heterogenicity of tumor in 3-dimension with spatial transcriptomics technologies. While the work is very interesting and would be quite beneficial to the community for cancer CNV detection, if authors could provide sufficient evidence to demonstrate the reliability of SlideCNA in resolving tumor heterogenicity in core biopsy. In my opinion, authors need to address following major concerns.
1) Baseline performance. Authors need to perform slide-seq and use SlideCNA on normal tissues and demonstrate homogeneous cell populations being detected. This study would provide an estimate on possible false discovery by SlideCNA. It is recommended to use at least three replicates from different patients.
2) RNA-seq and WES (or WGS) on bulk RNA and DNA from HTAPP-895-SMP-7359 and HTAPP-944-SMP-7479 should be conducted. Gene expression from RNA-seq and CNA analysis on WES/WGS should be conducted and correlation of expression alteration and CNA should be established. This result should be used to evaluate result from SlideCNA on slide-seq. Author only did WES on HTAPP-895-SMP-7359, which was shown in Fig. 2k.
3) The results presented in Figures 2k and 2i are not detailed enough and do not provide adequate granularity to support the conclusions made regarding the comparison of Slide-seq to snRNA-seq and SlideCNA to inferCNV. Authors need to provide more detail in explaining how the comparison was done and at what level results were compared. For example, it appears that "deletion" and "amplification" were shown as either whole chromosome or whole arm. How would SlideCNA or inferCNV determine whole arm or chromosome being gain or loss?
4) Tumor content in HTAPP-895-SMP-7359 and HTAPP-944-SMP-7479, should be determined with bulk WES/WGS and compared to number of malignant cells and normal cells derived from SlideCNA and inferCNV. This would provide another validation on the performance of SlideCNA and inferCNV.
5) The data used in the in-silico study came from the snRNA-seq of HTAPP-895-SMP-7359 and HTAPP-944-SMP-7479, which were also used in the slide-seq. Therefore, there is a huge bias in using same samples in both in-slico and validation study. I would suggest authors to use data set from a public source to perform in-silico study, for example Chen et al. (Nature Biotechnology, 2021, Sept.) has performed single cell sequencing benchmarking study with well-established reference samples (a pair of matched tumor/normal cell lines).
6) Both abstract, Background and Conclusion were too brief to provide insightful information. The authors should also address the limitations of SlideCNA and provide guidance on when it is and is not appropriate to use the toolkit.

**Comments to author:**

**Authors Response**

**Response to Reviewers**

We thank the Reviewers for their helpful commentary and suggestions to SlideCNA that we have taken into careful consideration to improve the reproducibility, user friendliness, and robustness of SlideCNA for greater potential impact. We have significantly revised the figures and the text, and added 7 new supplementary figures to accommodate these improvements.

**Response to Reviewer #1**:

*Zhang et. al present their novel computational method (SlideCNA) which estimates copy-number alterations (CNA) based on RNA expression captured in a spatial manner utilizing Slide-seq technology.*

*This is an interesting approach as it directly extends the functionality of current Slide-Seq experiments and therefore warrants eventual publication.*

*However, this current manuscript should be improved by a more rigorous curation and detailed (automated/reproducible) methodology of the chosen metrics, samples, reference set of non-malignant beads/cells and assessment of chromosomal-arm levels used in the final comparison between SlideCNA/InferCNV on Slide-Seq and non-spatially aware snRNA-Seq vs. WES/ABSOLUTE. In addition, the code and workflow itself (as deposited on Github) currently lack example data, vignettes and unit tests which makes assessing the robustness and validity of the code impossible and non-reproducible.*

We thank the Reviewer for their interest in this work and for recognizing SlideCNA’s potential to extend the functionality of current Slide-seq experiments. We are grateful for the Reviewer’s thorough and detailed review, helpful comments, and suggestions. Amongst other things, as detailed below, we have significantly improved our automation and reproducibility and provide the **respective analysis Jupyter notebooks as vignettes in a dedicated Github repository (**<https://github.com/dkzhang777/SlideCNA_Analysis>) with **accompanying example data** on Zenodo (<https://zenodo.org/records/10658097>).

Additionally, all the data used are now available on CELLxGENE, where samples can also be directly browsed and downloaded using the following link and selecting the respective Slide-seq or snRNAseq samples: https://cellxgene.cziscience.com/collections/a96133de-e951-4e2d-ace6-59db8b3bfb1d

*Major Concerns:*

1. *Comment #1 - Annotation of non-malignant cells in Slide-Seq.*
   a. *The current annotation of non-malignant cells is not 100% convincing. Please elaborate on the manual malignant/non-malignant annotation of SNN-based Seurat clusters within the Slide-Seq data, e.g., HTAPP-895-SMP-7359 (line 11, page 34). Could this be replicated in an automated and/or reproducible manner?*

We thank the Reviewer for raising the concern of annotation of our non-malignant cells and reproducibility. Following the Reviewer’s suggestion, we implement **an automated cell type annotation of Slide-seq data** (including the identification of malignant/non-malignant cells) **with RCTD** (Robust Cell Type Decomposition, <https://doi.org/10.1038/s41587-021-00830-w>) using the cell type annotations of the matching snRNA-seq data as reference (**Methods line 330 ff, Supplementary Fig. 13d**). This eliminated the need for manual assignment and affords greater reproducibility. In addition, this new annotation procedure also improved the non-malignant reference population for HTAPP-944-SMP-7479, such that reference transfer from HTAPP-895-SMP-7359 was no longer required.

We provide two Jupyter notebooks on Github to reproduce the final SlideCNA results (<https://github.com/dkzhang777/SlideCNA_Analysis>) from the raw Slide-seq counts matrix and the Slide-seq data after performing a bead decomposition procedure (described below) with TACCO.

b. *It is unclear how the top malignant/non-malignant clusters were chosen for the 'malignant gene-set' analysis (page 11; line 46-47). E.g., which DEGs an GO terms (and direction thereof) were defined as 'malignant'?*

This issue no longer pertains with our new annotation strategy, as described above.

c. *Fig. 2d detailing the DEGs between the CNA-clusters in HTAPP-895-SMP-7359 (where most non-malignant beads seem to fall into binned bead CNA cluster 3) show only three DEGs between the non-malignant and malignant CNA clusters. This may possibly indicate that the manual designation of these non-malignant beads may need some additional curation as other beads have likely also captured non-malignant cells and therefore rendering a differential expression analysis difficult unless the expression of genes fluctuates heavily within the malignant clusters.*

We thank the Reviewer for raising this point and agree that the differential expression testing of SlideCNA clusters could use improvement.

We have now cleaned up the Slide-seq data by semi-manually aligning matching H&E stains with Slide-seq data to remove non-overlapping spurious measurements and filter such that the fraction of beads with less than 100 counts did not exceed 35%. We re-annotated the Slide-seq data with RCTD to improve CNA calling and SlideCNA clustering. We also use a negative binomial generalized linear model for differential expression testing instead of a Wilcoxon rank sum test, because Slide-seq data are UMI-based (<https://satijalab.org/seurat/reference/findallmarkers>). Following these improvements, we identify a greater number of DEGs between each cluster in HTAPP-895-SMP-7359 (**Fig. 2e**). In HTAPP-944-SMP-7479, we see less and subtly different cluster-based significant DEGs, likely due to cell type mixing during bead capture, binning, and clustering, since the sample lacks discrete cell type zones (**Fig. 2k**).

To tackle the challenge of cell type mixing, we applied a new analysis strategy from our recently published TACCO framework (<https://doi.org/10.1038/s41587-023-01657-3>). In this approach, which we refer to as “bead decomposition”, each bead’s expression profile is partitioned into cell-type specific expression profiles based on its cell-type composition (as inferred during cell type annotation with RCTD). Thereby, for each bead we obtain one “pure” expression profile for each contributing cell type. Using these “pure” expression profiles for our SlideCNA analysis, we identified more DEGs between clusters (**Supplementary Fig. 12**). Since the “bead decomposition” approach might also introduce biases, we did not introduce it as a SlideCNA default, but describe it as a potential improvement (**Results line 203-216, Methods line 474**) and provide a relevant Jupyter notebook vignette (<https://github.com/dkzhang777/SlideCNA_Analysis>.

d. *Furthermore, the authors transplanted these 'reference' non-malignant beads from HTAPP-895-SMP-7359 onto HTAPP-944-SMP-7479 (with spatial offset) as the latter sample lacked intrinsic 'non-malignant' beads (according to the manual annotation).
   As these non-malignant cells are the baseline reference and thereby the foundation against which CNA-scores are determined with SlideCNA, I would have preferred a more thorough (and reproducible) investigation into the confidence of these 'non-malignant' cells.*

We thank the Reviewer for this important comment. As noted above, with our improved annotation strategy, the reference transplantation is no longer required.

Nevertheless, we do note that the CNAs identified in HTAPP-944-SMP-7479 with the reference transfer (in the original manuscript) are mostly consistent with those in the new, RCTD-annotated HTAPP-944-SMP-7479 Slide-seq sample without a reference transfer (current manuscript) (**Fig. R1**). This suggests that using a non-malignant reference transfer from HTAPP-895-SMP-7359 does not significantly alter CNA calls.

**Fig. R1:** Comparison of SlideCNA results for HTAPP-944-SMP-7479 Slide-seq data with (**a**) and without (**b**) reference transplant. Annotation of malignant and non-malignant populations on spatial axes (i), SlideCNA-defined clusters of binned beads (ii), and SlideCNA heat maps from binned beads (iii).

e. *Could the authors also add additional histopathological information based on H&E on these samples supporting their decision-making?*

We thank the Reviewer for this great suggestion. We now provide H&E stains of adjacent sections for our four Slide-seq samples (**Fig. 2c,i and Supplementary Figs. 8c,j and 13a**) with pathologists’ annotations highlighting regions that are dominated by malignant cells or non-malignant tissue components, respectively. These show overall concordance with our analysis.

f. *Adding DAPI or similar staining of the tissue section to Fig. 2 (rather than just bead signal in Fig. 2a) would also clarify tissue structures. It is currently rather unclear what the 'white space' is and to what the sparse outer lying beads signals refer to.*

We thank the Reviewer for this suggestion. While we unfortunately could not perform DAPI staining on the same tissue section that was used to generate the Slide-seq data, we have added H&E stains of adjacent sections along with expert pathologist annotations (**Fig. 2c,i** and **Supplementary Figs. 8 and 13**).

As for the ‘white space’ and sparse outer lying bead signal, we now perform CNA calls after **aligning the H&E image with the Slide-seq signal** and **removing the outer lying beads that do not overlap the tissue** and most likely contained spurious signal from ambient RNA, similar to typical QC of other spatial transcriptomics data, such as Visium (**Methods line 306-308**).

2. *Comment #2 - Selection and importance of spatial and expression profile weights in calculating the pseudo-distance.*
   a. *The authors should provide more detailed explanation of how tuning parameters are chosen. The authors have used k=55 and k=1 for weighing the spatial and expression profiles respectively to calculate the pseudo-distance matrix on which subsequent bead-clustering is performed.*

*Currently, there is no rationale for these chosen values beyond selecting for spatially close beads over (divergent) expression profiles. Could the authors explain their chosen weights (k=55 and k=1) and the dependency of SlideCNA for various iterations of k (e.g., bootstrapping of CNA clusters with various employed weights) to give users a feeling for which weights would be preferable in their samples and how much this affects the robustness of CNA clusters.*

We thank the Reviewer for raising this important point. Following the Reviewer’s suggestion, we now perform **bootstrapping of SlideCNA’s parameters** on HTAPP-895-SMP-7359. SlideCNA uses the parameters *pos_k* (spatial parameter, default=55) and *ex_k* (expression parameter, default = 1) to control the relative weights of each component when binning. Since the two parameters are relative (**Methods**), we only need to bootstrap one to change their ratio. We examined binning and clustering results when SlideCNA uses relatively low, moderate, and high spatial weights (*pos_k* = 0.01, 0.5, and 100, respectively; *ex_k* = 1) (**Supplementary Fig. 14, line 382**). Overall, CNA patterns were quite stably maintained across this broad range of parameters, but a higher spatial weight yielded cleaner CNA patterns and a more discrete spatial patterning of CNA-based clusters, justifying our default parameter choice.

3. *Comment #3 - Transplantation of non-malignant cells between MBC samples.*

*The approach of cut and pasting beads to another samples raises questions as to whether this introduces biases from batch effects.*

*Fig 2i reveals more uniform expression differences (DEGs) between the transplanted beads vs. the host malignant beads (CNA cluster 1 and 2 vs. 3) compared to the CNA cluster comparison within the donor sample in which I had expected more (or similar) DEGs between the malignant/non-malignant cluster. This might indicate that there are truly no non-malignant cells in cluster 3 (original beads of host) and that a clean biological comparison has been performed, or that there are also other subtle (both non-uniform and uniform) batch-effects in place which cause an increase of DEGs such as sample-specific batch-effects.*

*The authors claim that the transplantation of these non-malignant cells between Slide-Seq samples is a viable approach (page 12; line 4-9) with only a reference to the GitHub repository of their previous methodology (inferCNV). This reviewer is unable to find the supporting evidence for this statement within this repository such as an inter-sample comparison between the expression profiles of non-malignant cells in multiple Slide-Seq (or other scRNA-Seq) experiments which altogether rule out the occurrence of sample-specific batch-effects.*

*Could the authors indicate where exactly within this repository such a comparison would be located and/or provide additional references or supplemental data supporting this claim; as sample-specific batch effects have long been a known factor in the analysis of sequencing data, including scRNA-Seq.*

We thank the Reviewer for raising the question of sample-specific batch effects. As noted above, with our new annotation strategy, reference transfer is no longer necessary.

Nevertheless, we regret the confusion caused by our lack of detail. With respect to InferCNV, we were referring to a common practice to compile a reference of healthy cells across different samples for InferCNV. Here, the rationale is that healthy cell profiles are typically consistent across individuals (and should not contain substantial recurrent CNAs) and thus can be pooled to generate a robust reference. This is detailed in the InferCNV input example (<https://github.com/broadinstitute/inferCNV/wiki/File-Definitions>), where non-malignant cells are from patient MGH54, while the malignant cells are from patient MGH36. (This is no longer relevant, however, to our manuscript.)

4. *Comment #4 - Overall comparison of copy-number profiles between methodologies (InferCNV/SlideCNA) and technologies (Slide-Seq, snRNA-Seq and WES).*
   a. *The authors should conduct a more rigorous comparison to copy number alteration data from an established technology. The comparison of copy number profiles as estimated by ABSOLUTE on whole-exome sequencing (WES) inferCNV/SlideCNA on the Slide-seq and the snRNA-seq (without spatial parameters) lacks a reproducible methodology. I.e., what was the schematic to 'manually' consider arms to be deleted or amplified (Fig 2i-k) from the data of (among others) Fig. S4 and how robust is this calling?*

*For instance, S4E might also indicate an allelic balance/CN-neutral category for 2p yet Fig. 2i has 2p listed Deleted' under the WES category (which corresponds with SlideCNA). How exactly to interpret bead-wise Slide-seq/snRNA-seq (Fig. S4a-d) CNA-scores for a general sample-wise arm-level estimate is even more prone to personal bias if manual annotation is performed.*

*Furthermore, was this calling performed in a blinded manner (e.g., WES results were kept from assignee(s) prior to calling arms in snRNA/Slide-seq) or could there be (albeit unknown) bias in manual calling?*

*For instance, could the authors perhaps use CNA-scores of similar genomic windows to estimate the (per arm) similarity between the compared approaches rather than visual inspection and annotation?*

We thank the Reviewer for this excellent comment and agree that a more quantitative assessment is required. Following the Reviewer’s comment, we now computed Spearman’s rank correlation coefficient between average CNA scores per chromosomal arm for each pair of combination of data modality and CNA calling method (**Methods line 464 ff, Results line 173 ff, Fig. 2o,p and Supplementary Fig. 8q,r**). We additionally compared the numeric average CNA scores between SlideCNA, Infercnv, and CopyKAT applied to Slide-seq and snRNA-seq data (**Fig. 2m,n** and **Supplementary Fig. 8o,p**), and, similarly, to WES calls with ABSOLUTE ( **Fig. 2m,o and Supplementary Fig. 9e**).

b. *In addition, it is unclear how SlideCNA was exactly performed on the snRNA-seq "with non-spatial parameters" (page 6; line 11-13). Does this mean that only X_expression (genes x beads with k_expression =1) was given with an empty X_spatial with _kspatial=0 whilst otherwise implementing the same algorithm (page 12-14; SlideCNA) or was another implementation used?*

We regret the lack of clarity on this point. To apply SlideCNA to snRNA-seq data “with non-spatial parameters” means that we set the spatial parameter to False in run_slide_cna(). This removes the *k_spatial* parameter and spatial distance matrix from the pseudospatial distance calculation effectively making it a standard expression matrix. This also removes spatial plotting from SlideCNA. We clarify this in the revised (**Methods** **line 385**).

5. *Comment #5 - Assessment of code/workflow*
   a. *The package (*[*https://github.com/dkzhang777/SlideCNA/*](https://github.com/dkzhang777/SlideCNA/)*) is currently missing a vignette, example data and unit tests to determine the robustness of the code and functions itself. For instance, the package does not currently satisfy the minimal requirements to be incorporated within BioConductor which is a good benchmark to assess minimal reproducibility and robustness.*

We thank the Reviewer for this important guidance. Following the Reviewer’s suggestion, we **added vignettes** of (**1**) the SlideCNA workflow using RCTD annotations and (**2**) the SlideCNA workflow with RCTD annotations and bead decomposition with TACCO for HTAPP-895-SMP-7359. These are available at <https://github.com/dkzhang777/SlideCNA_Analysis>. In the first vignette, we supply the HTAPP-895-SMP-7395 Slide-seq filtered raw counts matrix and metadata, perform RCTD annotations with matching snRNA-seq data, and run SlideCNA. In the second vignette, we supply the RCTD-annotated Slide-seq data, run TACCO for bead decomposition, then run SlideCNA.

Example data to go with the vignettes is now provided through Zenodo (<https://zenodo.org/records/10658097>).

We have also added tests to the SlideCNA code and have submitted it to CRAN (pending approval, see screenshot of confirmation e-mail), with these updates available on <https://github.com/dkzhang777/SlideCNA>.

b. *Could the authors add this to the package to allow for a review of the workflow/code and to assess the user-friendliness? For instance, the description on how the metadata should be given (md in run_slide_cna()) is currently only available on the README rather than described within the function with accompanying example data.*

*As such, this reviewer could currently not perform, reproduce nor assess the validity of the package and SlideCNA.*

We greatly appreciate this feedback and have subsequently revised the SlideCNA code to improve usability and reproducibility. In the **Jupyter notebook vignettes** of SlideCNA (<https://github.com/dkzhang777/SlideCNA_Analysis>), we provide example data of an example workflow, which improves user-friendliness and serves as a reference for how one might format the inputs. We also added a description of the parameters including ‘md’ of run_slide_cna() within the function and README. Lastly, we have updated the code documentation in accordance with CRAN standards to improve usability with the goal of also making SlideCNA available via conda.

6. *Minor concerns*
   a. *(Page 11; Line 14) Annotation of cell-types using SingleR.*

*Please specify the used reference (e.g., Human Primary Cell Atlas (Mabbott et al. 2013) used to annotate cell-types with SingleR.*

We regret this omission. We used the Human Primary Cell Atlas (Mabbott et al., 2013), ENCODE (ENCODE Project Consortium, 2012), and BLUEPRINT (Aran D, Looney AP, Liu L et al., 2019) to annotate cell types with SingleR. We then unified these cell types using a cell-type nomenclature unification list (**Methods line 314 ff**).

b. *De-novo reference bead selection in Slide-Seq (M&M).*

*The term "bead cluster" (i.e., SNN-based clusters from the Seurat workflow on the Slide-seq data) can be seen as confusing with the (later defined) pseudo-distance calculated bead clusters in the CNA analysis and would benefit from a distinct term to distinguish the two (e.g., SNN-derived Slide-Seq bead cluster).*

Since we have now replaced the SNN-based cluster *de-novo* reference bead selection in Slide-seq with RCTD-based annotations, we no longer need to use this term at all and removed it from the manuscript.

c. *Suppl. Fig S2, S3 and S6*

*Typo in y-axis title of boxplot; "ber bead" rather than "per bead".*

Thank you. These typos were fixed (**Supplementary Figs. 2, 3, and 13**).

d. *X-ticks Suppl. Fig. 6c-d.*

*Why are there five x-axis tick-lines if it is a single group/boxplot?*

Thank you. The extra tick lines were removed (**Supplementary Fig. 13b**).

e. *Definition of non-malignant clonal cluster.*

*As hierarchical clustering on CNA-scores is only performed on the malignant bead-bins and all non-malignant bead-bins are defined as a separate category (rather than through clustering), it might be clearer to a reader to annotate this non-malignant group not as a separate cluster (with incremental/decreasing cluster ID/name) but simply as 'Non-malignant' or similar to emphasize that these were not defined as a group by clustering but through other annotation.*

We regret our lack of clarity on this procedure. We actually **do cluster both malignant and non-malignant binned beads together, not separately.** However, to determine the number of clusters as input for the *cut_tree* function which assigns clusters, we determine the best number of clusters for the malignant binned beads only, then we added one (representing one non-malignant cluster) to arrive at the total number of clusters. Next, we performed hierarchical clustering on all binned beads together and use the total number of clusters (as described above) to cut the hierarchical tree yielding the final presented cluster assignments. We describe this more clearly in the revised **Methods** (**line 397 ff**). Please note, that in its current setup for clone calling, SlideCNA will suggest a minimum of three clusters and the user has to discern based on the heatmap if the CNA patterns sufficiently support these clusters as biologically meaningful “clones”. We also describe this for sample HTAPP-944 (**line 150 ff**).

f. *Missing M&M on STARCH*

*Please describe the usage, version and any non-default parameters used in the STARCH analysis within the Methods section. Currently, this is only present within the results (Page 7; line 11-29).*

Unfortunately, we had to remove STARCH from our analysis. Although we wanted to display results of STARCH to show SlideCNA’s performance relative to another CNA calling method for ST data, we ran into an implementation error which was not resolved through varying installations of python. This issue is well-documented on Github and has been raised by numerous users and has not yet been resolved (<https://github.com/raphael-group/STARCH/issues/4>). Given this and the fact that STARCH CNA profiles on our old Slide-seq data for HTAPP-895-SMP-7359 and HTAPP-944-SMP-7479 with a reference transfer were not promising (potentially because it wasn’t developed for the sparsity and resolution of Slide-seq data). (**Fig. R2**), we decided to remove STARCH.

**Reviewer Fig. 2:** STARCH CNA profiles and spatial CNA-based cluster assignments for the old, uncleaned (pre-review) versions of HTAPP-895-SMP-7359 (**a,b**) and HTAPP-944-SMP-7479 with reference transfer (**c,d**) Slide-seq data.

g. *Please double-check the spelling within the SlideCNA code. E.g., the main function (run_slide_cna) has a typo within its required parameters (OUPUT_DIRECTORY).*

Thank you. We fixed these typos in the SlideCNA code (<https://github.com/dkzhang777/SlideCNA/>).

h. *Authors claim that their previous method (InferCNV) is the current gold standard for CNA-detection in scRNA-seq (page 6, line-26). Do the authors have additional support for this claim?*

*E.g., how does it compare with the current adoptions of the widely-used CopyKAT method for scRNA-Seq which showed closer approximation to bulk reference DNA copy number profile (at 220Kb genomic resolution) in their comparison to inferCNV? (Gao R, Bai S, Henderson YC, Lin Y, Schalck A, Yan Y, Kumar T, Hu M, Sei E, Davis A, Wang F, Shaitelman SF, Wang JR, Chen K, Moulder S, Lai SY, Navin NE. Delineating copy number and clonal substructure in human tumors from single-cell transcriptomes. Nat Biotechnol. 2021 May;39(5):599-608. doi: 10.1038/s41587-020-00795-2. Epub 2021 Jan 18. PMID: 33462507; PMCID: PMC8122019)*

We revised our statement that InferCNV is the “gold standard” to being “one current method for scRNA-seq”. In addition, we added an implementation of CopyKAT and demonstrate that CopyKAT and InferCNV applied to snRNA-seq data are mostly concordant (as is SlideCNA), except for sample HTAPP-880-SMP-7179, for which CopyKAT results are discordant to those of InferCNV and SlideCNA for both snRNA-seq and Slide-seq, likely due to CopyKAT’s mis-annotation of a non-malignant reference population (which CopyKAT does in an automated manner and does not accept this annotation as user input). CopyKAT run on Slide-seq data, however, produces discordant CNA calls compared to all other method-data combinations (**Fig. 2m,n** and **Supplementary Figs. 8m,n and 11**).

**Response Reviewer #2:**

*Zhang et al. developed SlideCNA, a toolkit that infers copy number alteration (CNA) from slide-seq spatial transcriptomics data. Similar to inferCNV, SlideCNA uses contiguous chromosomal expression changes to infer copy number alterations. Thus, both tools were based on assumption that alteration of expression abundance was primary due to change in gene dosage. Using snRNA-seq of metastatic breast cancer tumor core biopsy from two patients, authors constructed an in-silico slide-seq data set to demonstrate the performance of SlideCNA. Finally, authors applied SlideCNA on the actual slide-seq on a separated core biopsies from same patients.*

*In general, it is a very challenging task to infer CNA with altered gene expression in cells. The accuracy and granularity achieved with these types of method were very limited (Slyper M., et al. Nature Medicine 26, 792-802 (2020)). SlideCNA does not offer any significant innovations and is primarily an improved version of inferCNV. Nevertheless, it would be helpful to understand the heterogenicity of tumor in 3-dimension with spatial transcriptomics technologies. While the work is very interesting and would be quite beneficial to the community for cancer CNV detection, if authors could provide sufficient evidence to demonstrate the reliability of SlideCNA in resolving tumor heterogenicity in core biopsy. In my opinion, authors need to address following major concerns.*

We thank the Reviewer for their interest in this work and for highlighting its potential benefits to the community. We appreciate the Reviewer’s constructive assessment and have done our best to address the Reviewer’s concerns.

*Major concerns:*

1. *Comment #1 - Baseline performance. Authors need to perform slide-seq and use SlideCNA on normal tissues and demonstrate homogeneous cell populations being detected. This study would provide an estimate on possible false discovery by SlideCNA. It is recommended to use at least three replicates from different patients.*

We thank the Reviewer for this important comment. We agree that addressing false discoveries is indeed important.

First, to assess possible false discovery in each analysis we not only call CNAs in the malignant cells, but also in the normal cells within the same sample and display these calls within the same heat map (on snRNAseq in **Supplementary Figs. 9c,d, 10c,d** on Slide-seq in **Fig. 2f,l,** and **Supplementary Fig. 7b,c,g,h (panel iii)**). These internal controls typically show “random” signal or, in some cases non-random signals that are, however, different from the signal in the malignant cell population. These internal controls in each run allow us to assess whether signals in the malignant cells might be false positives (if they also appear in the normal cells).

Second, we now include four more Slide-seq samples from two new patients/metastases in our analysis, HTAPP-878-SMP-7149 and HTAPP-880-SMP-7179 (**Supplementary Fig. 7**), where we have two replicates (consecutive sections) for each patient/metastasis, which show very high agreement adding further confidence in the correctness of our CNA calls. In total we now include Slide-seq data from four different patients, two of which have technical replicates from consecutive sections.

Finally, we also followed this reviewer’s suggestion of running SlideCNA on healthy tissue. To this end, we used a well-established mouse brain sample published with the original Slide-seq publication (<https://singlecell.broadinstitute.org/single_cell/study/SCP815/highly-sensitive-spatial-transcriptomics-at-near-cellular-resolution-with-slide-seqv2#study-summary>). This sample has a distinct cell type and signal density structure creating a situation prone to call false positives (**Fig. R3 a**). Because in healthy tissue it is not clear which cells/beads should be used as reference (non-malignant cells in tumor tissue), we randomly designated 25% of the beads as “non-malignant” reference and the rest as “malignant” (**Fig. R3 b**). The resulting SlideCNA heatmap (**Fig. R3 c**) showed mostly random noise and incoherent signals as expected for a tissue without strong real CNAs. Please note, that in its current setup for clone calling (**Methods line 397 ff**) SlideCNA will suggest a minimum of three clusters (2 “malignant” and 1 for healthy cells) and the user has to discern based on the heatmap if the CNA patterns sufficiently support these clusters as “clones”. We also describe this for sample HTAPP-944 (**line 150 ff**). Here, in the healthy tissue identified clusters don’t show clear coherent differences, but even intermixing of the designated “healthy” beads – as expected.

Even though it is technically possible to run SlideCNA on healthy tissues, it does not represent a valid use case since the reference is not naturally defined and has to be chosen artificially for example by random selection.

| a | c |
| --- | --- |
| b |  |
| **Reviewer Fig. 3**: SlideCNA applied to Slide-seq data of healthy mouse brain tissue of the hippocampal region. a) Slide-seq beads plotted in space and colored by their cluster assignment as produced by Leiden clustering using the Seurat package. b) Same as a) but colored by random assignment of beads as either malignant or non-malignant, with non-malignant beads serving as reference in the SlideCNA analysis. c) Resulting SlideCNA heatmap. | |

2. *RNA-seq and WES (or WGS) on bulk RNA and DNA from HTAPP-895-SMP-7359 and HTAPP-944-SMP-7479 should be conducted. Gene expression from RNA-seq and CNA analysis on WES/WGS should be conducted and correlation of expression alteration and CNA should be established. This result should be used to evaluate result from SlideCNA on slide-seq. Author only did WES on HTAPP-895-SMP-7359, which was shown in Fig. 2k.*

We thank the Reviewer for this suggestion. While matching WES and bulk RNA-seq data for every sample would be ideal, unfortunately, this was not possible for any of the other samples because they were used up completely for Slide-seq and snRNA-seq, as these were metastatic biopsies with limited material. We additionally validate SlideCNA by including an additional CNA calling method (CopyKAT) and introducing numeric comparisons across different combinations of pairs of methods (SlideCNA, InferCNV, CopyKAT, ABSOLUTE) and data modalities (snRNA-seq, Slide-seq, WES) (**Fig. 2o,p, Supplementary Fig 8o,p**) across a total of four metastases (2 additional tissue samples, including replicates, ABSOLUTE and WES only available for HTAPP-895-SMP-7359). We now show the comparison between RNA-seq-based SlideCNA calls and the WES calls for HTAPP-895-SMP-7359 in the same figure (**Supplementary Fig. 9e** and **Reviewer Fig. 2**), and compare the CNA profiles quantitatively (**Fig. 2o,p**).

3. *The results presented in Figures 2k and 2i are not detailed enough and do not provide adequate granularity to support the conclusions made regarding the comparison of Slide-seq to snRNA-seq and SlideCNA to inferCNV. Authors need to provide more detail in explaining how the comparison was done and at what level results were compared. For example, it appears that "deletion" and "amplification" were shown as either whole chromosome or whole arm. How would SlideCNA or inferCNV determine whole arm or chromosome being gain or loss?*

We thank the Reviewer for this comment, which was also raised by **Reviewer 1**. To improve granularity and reproducibility, now use compute Spearman’s rank correlation coefficient between the profiles of average CNA scores per chromosomal arm from each pair of method/modality combination **(Methods line 464 ff, Results line 173 ff)**, with three (four) methods, SlideCNA, Infercnv, and CopyKAT (and ABSOLUTE, for HTAPP-895-SMP-7359) (**Fig. 2m-p** and **Supplementary Fig. 8o-r**). We no longer rely on any manual calling.

4. *Tumor content in HTAPP-895-SMP-7359 and HTAPP-944-SMP-7479, should be determined with bulk WES/WGS and compared to number of malignant cells and normal cells derived from SlideCNA and inferCNV. This would provide another validation on the performance of SlideCNA and inferCNV.*

We agree that determining tumor content is important. As noted above, it was not possible to generate WES/WGS for both samples due to complete sample usage, and moreover, the WES data for HTAPP-895-SMP-7359 was generated from an adjacent core of the same metastasis due to sample limitations. Instead, following the suggestion of **Reviewer 1**, we analyzed H&E images from an immediate adjacent section for each sample, with expert pathologist annotations, which allow for a rough estimate of tumor content. Those showed good qualitative agreement with SlideCNA (**Fig. 2c,i** and **Supplementary Figs. 8c,j** and **13a**).

5. *The data used in the in-silico study came from the snRNA-seq of HTAPP-895-SMP-7359 and HTAPP-944-SMP-7479, which were also used in the slide-seq. Therefore, there is a huge bias in using same samples in both in-silico and validation study. I would suggest authors to use data set from a public source to perform in-silico study, for example Chen et al. (Nature Biotechnology, 2021, Sept.) has performed single cell sequencing benchmarking study with well-established reference samples (a pair of matched tumor/normal cell lines).*

We thank the Reviewer for this important point. To address this concern, we analyzed scRNA-seq data from two primary breast tumors (Wu et al., 2021, <https://doi.org/10.1038/s41588-021-00911-1>). SlideCNA was effectively able to separate the two cancers based on the CNA calls and reliably call CNAs similar to those obtained by InferCNV in the original study (**Supplementary Figs. 4-6**). We prioritized using this dataset over the one suggested by the Reviewer, because, unlike Chen et al’s cell line data, Wu et al is tumor data and already included CNA calls that let us select sufficiently different samples and could serve as ground truth.

6. *Both abstract, Background and Conclusion were too brief to provide insightful information. The authors should also address the limitations of SlideCNA and provide guidance on when it is and is not appropriate to use the toolkit.*

We thank the Reviewer for this perspective. In the abstract, we now emphasize the importance of the binning approach to overcome sparsity while maintaining spatial structure (**line 48**). In the background, we more thoroughly describe the gap in the field of applying existing CNA detection methods to spatially dense, transcriptionally sparse spatial transcriptomics data and thus the importance of creating a method that overcomes Slide-seq’s sparsity and cell type mixing during the bead generation process while leveraging spatial data to inform subclonal structure and contextualize CNA patterns (**line 76 ff**). Supporting this claim, in our method benchmarking (**Fig. 2o,p** and **Supplementary Fig. 8q,r**) we show that InferCNV and CopyKAT, two methods developed for scRNAseq data, show high concordance on the snRNAseq data but lose significantly in concordance when run on Slide-seq data, while SlideCNA maintains high correlations. This indicates that the introduction of a dedicated method for Slide-seq-like spatial transcriptomics data is warranted.

We appreciate the suggestion to provide readers with appropriate use of the toolkit and have now added the limitations and guidelines of SlideCNA usage in the **Conclusions (line 218 ff)**.

**Second round of review**

**Reviewer 1**

---

**Reviewer 2**

Authors have done good jobs in addressing mine and other reviewers' comments. I would recommend accepting this draft for publication on your journal. Thank you for having me!
